# Intrastriatal Grafting of Chromospheres: Survival and Functional Effects in the 6-OHDA Rat Model of Parkinson's Disease

**DOI:** 10.1371/journal.pone.0160854

**Published:** 2016-08-15

**Authors:** Alejandra Boronat-García, Marcela Palomero-Rivero, Magdalena Guerra-Crespo, Diana Millán-Aldaco, René Drucker-Colín

**Affiliations:** Instituto de Fisiología Celular, Departamento de Neuropatología Molecular, Universidad Nacional Autónoma de México, Circuito Exterior s/n, Ciudad Universitaria, Ciudad de México, México; Hudson Institute, AUSTRALIA

## Abstract

Cell replacement therapy in Parkinson’s disease (PD) aims at re-establishing dopamine neurotransmission in the striatum by grafting dopamine-releasing cells. Chromaffin cell (CC) grafts produce some transitory improvements of functional motor deficits in PD animal models, and have the advantage of allowing autologous transplantation. However, CC grafts have exhibited low survival, poor functional effects and dopamine release compared to other cell types. Recently, chromaffin progenitor-like cells were isolated from bovine and human adult adrenal medulla. Under low-attachment conditions, these cells aggregate and grow as spheres, named chromospheres. Here, we found that bovine-derived chromosphere-cell cultures exhibit a greater fraction of cells with a dopaminergic phenotype and higher dopamine release than CC. Chromospheres grafted in a rat model of PD survived in 57% of the total grafted animals. Behavioral tests showed that surviving chromosphere cells induce a reduction in motor alterations for at least 3 months after grafting. Finally, we found that compared with CC, chromosphere grafts survive more and produce more robust and consistent motor improvements. However, further experiments would be necessary to determine whether the functional benefits induced by chromosphere grafts can be improved, and also to elucidate the mechanisms underlying the functional effects of the grafts.

## Introduction

Parkinson´s disease (PD) is a neurodegenerative disorder characterized by non-motor and motor symptoms. The latter are the principal targets of current pharmacological therapies, as they are the most disabling for the patients. The physiopathology of PD motor symptoms is associated with the loss of dopaminergic neurons in the substantia nigra pars compacta (SNpc), which project to the striatum and release dopamine [1]. As a consequence, the dopamine levels in the striatum decrease, leading to the classical motor alterations in PD, such as resting tremor, muscle stiffness, paucity of voluntary movements and postural instability [2]. Levodopa is the gold standard choice for treating the motor symptoms, but levodopa doses have to be increased with the progress of the disease, inducing motor complications and side effects (e.g. dyskinesias) [3]. As an alternative, cell replacement therapy was proposed during the eighties decade, and was aimed at providing an external source of dopamine in the striatum by grafting dopamine-releasing cells [4]. Chromaffin cells (CC) from the adrenal gland were one of the first types of grafted cells to be tested, due to their ability to release catecholamines and other neuroactive molecules [5]. In animal models of PD, CC grafting was shown to induce moderate but highly variable motor improvements [5–7], even though only 1% of CC are able to release dopamine [8]. In the clinic, however, these cells have not yielded satisfactory results, as their grafting in human patients produced only partial motor improvements [9] that were only transitory and highly variable among subjects [10]. Furthermore, high morbidity and mortality were associated with this grafting procedure, precluding further use of CC grafts [10].

Other types of cells have been tested in animal models as possible sources for cell replacement therapy, including fetal ventral-mesencephalic cells [11–13], and more recently embryonic and induced-pluripotent stem cells [14]. These cells can acquire a dopaminergic phenotype and are considered the most promissory alternative cell sources so far, as they have been shown to survive for longer periods of time than CC and to induce more robust motor improvements [14]. However, the use of these cells involves other important problems, including ethical and technical concerns related to the use of fetal-derived cells (e.g. fetal ventral-mesencephalic cells) or the tumorigenic potential associated with the use of proliferative cells [15–17]. These issues have limited their therapeutic application. Accordingly, it has been suggested that the search for an ideal source for cell replacement therapy in PD must be guided by the following requirements [18]: to be from human origin, to have the possibility of differentiating in large proportions into a dopaminergic phenotype (especially by showing the properties of SNpc neurons) and to produce significant motor improvements after being grafted in animal models of PD without any side effects, including tumor development. In addition, it would be preferable that the cell source is easy to obtain and to maintain in culture.

Recently, in an effort to isolate progenitor-like cells from adult adrenal medulla, Ehrhart-Bornstein and coworkers developed an isolation cell-culture protocol, which permits the expansion of progenitor-like cells from the adrenal medullae of postnatal mice [19], adult bovines [20] and humans [21]. Under low-attachment conditions, these progenitor-like cells aggregate and grow as spheres, named chromospheres. Chromosphere cells have a limited capacity of self-renewal and form clonal secondary spheres in culture [19,20]. Furthermore, chromosphere cells express increased mRNA levels of sympatho-adrenal and neuronal progenitor cell markers such as Nestin, Mash1, Vimentin, Musashi-1, as well as markers of neural crest cells (e.g. Sox1 and Sox10) [19,20,22]. Additionally, a significantly higher population of chromosphere-derived cells has been shown to acquire a dopaminergic phenotype and to extend projections as compared with CC [22].

Due to their favorable properties, chromospheres could represent a promissory source for cell replacement therapy in PD. Therefore, in this study we examined some of the molecular and functional properties of bovine-derived chromospheres *in vitro* and tested their effects following their grafting in the striatum of a rat model of PD.

## Materials and Methods

### Animals

Adult male Wistar rats (250–280 g) used in this study were kept under a 12 h light/dark cycle with *ad libitum* access to food and water. Rats were obtained from the Institute´s animal facility. All animal procedures were carried out following protocols approved by the Committee of Animal Use and Care of the Instituto de Fisiología Celular (Permit number: RDC1114) from the Universidad Nacional Autónoma de México, which are based on national and international standards.

### CC- and chromosphere- culture

The cell culture protocol employed for chromosphere formation was described previously [20–22]. Adrenal glands from adult cattle were freshly obtained from the local slaughterhouse (“Rastro Frigorífico La Paz”, Los Reyes Acaquilpan, Mexico) and transported to the laboratory in ice-cold d-PBS buffer (Sigma-Aldrich) supplemented with 2% penicillin/streptomycin solution. Prior to chromaffin and chromaffin progenitor-like cell isolation, adrenals were shortly put in 70% ethanol and connective tissue was removed. Adrenals were flushed several times with d-PBS through the central vein to remove any remaining blood. Next, d-PBS supplemented with 0.3% type II collagenase (Sigma-Aldrich) and 30 units/ml DNase I (Sigma-Aldrich) was injected through the central vein, and the tissue was then incubated at 37°C for 40 min, followed by a second injection and incubation for 20 min. Cells from the adrenal medulla were separated by mechanical dissociation, sieved through a ~300 μm chiffon two times, and then two more times through 100 and 70 μm cell strainers (Becton Dickson), and finally washed with d-PBS three times. Cells were then cultured in adherent culture flasks (Sarstedt) using DMEM/F12 (Gibco) supplemented with 10% steroid-free FBS (Charcoal-Dextran treated, Hyclone) and 2% penicillin-streptomycin, and kept overnight in an incubator with humidified atmosphere (95% air, 5% CO_2_) at 37°C. Afterwards, chromaffin and progenitor-like cells were separated from other cells types by differential plating as previously described [22]; briefly, cells were detached from the culture flasks and placed on a new adherent flask and incubated for 2 h, after which the supernatant containing the chromaffin and chromaffin progenitor-like cells was recollected and placed in a new adherent flask for 1 h, repeating this step one additional time. Finally, for chromosphere formation and expansion, isolated cells were seeded in ultra-low attachment culture flasks (Sarstedt) with Neurobasal medium (Gibco) containing 2% B27-supplement (Gibco), 1% penicillin/streptomycin, 2 mM L-glutamine (PAA laboratories) and 20 ng/ml basic fibroblast growth factor (bFGF, Sigma-Aldrich). For CC culture, after differential plating isolated cells were seeded in DMEM/F12 medium supplemented with 10% steroid-free FBS and 1% penicillin/streptomycin in adherent dishes. Fresh medium was added every two days.

### HPLC-ED measurements of *in vitro* catecholamine release

Three or 9 days after cell dissociation, CC and chromospheres were seeded at a density of 30,000 cells per well in 24-well plates previously treated with poly-L-Lysine (4 μg/ml) and laminin (5 μg/ml) (Sigma-Aldrich) to enhance cell adhesion, and incubated for 1 h in culture medium to allow the cells to adhere to the substrate. The culture medium was then substituted by 400 μl of physiological solution (140 mM NaCl, 2.4 mM KCl, 1 mM MgCl, 10 mM HEPES, 10 mM Glucose, 1.25 mM NaH_2_PO_4_, 2 mM CaCl_2_, pH 7.4), and the cells were incubated for 5 minutes at 37°C in a humidified atmosphere (95% air, 5% CO_2_) in the complete absence of light. The extracellular solution was recollected and supplemented with 0.01 M perchloric acid (HClO_4_) to avoid catecholamine oxidation. Samples were analyzed using HPLC with electrochemical detection (HPLC-ED). Calibration standard samples were diluted in the same physiological solution with 0.01 M perchloric acid. The mobile phase consisted of HPLC-grade water containing 0.15 M NaH_2_PO_4_, 0.5 mM EDTA, 0.5 mM sodium octylsulfate and 12.5% (v/v) methanol. The pH was adjusted to 3.8 with 1 M perchloric acid. The mobile phase and samples were filtered prior to use. 20 μl of each sample were injected for analysis using a Rheodyne 734 auto injector (Gilson Inc, Middleton USA). ED was performed using an LC-3C detector (BAS) prototype (750 mV vs Ag/AgCl) glassy carbon electrode. The 305-Gilson pump provided a flow rate of 1 ml/min through the column (nucleosil 5 C18, ODS, 100A 150 x 4.6 mm) and guard column. The final concentration of catecholamines is expressed in nmol.

### 6-OHDA nigro-striatal lesion and cell transplantation

To generate a unilateral PD model, adult male Wistar rats (250–280 g body weight) were intraperitoneally (I.P.) anesthetized with ketamine/xylazine (100 and 10 mg/kg, respectively), and standard sterile-surgical and stereotaxic procedures were employed for 6-OHDA infusion [23]. Anesthetized animals were mounted on a stereotaxic device and normal saline solution (0.5 μl total volume) supplemented with 40 μg 6-OHDA and 0.02% ascorbic acid was injected into the left SNpc (- 4.7 mm AP, ± 1.6 mm ML, -8.2 mm DV, with respect to Bregma 0). For cell transplantation 18 days after 6-OHDA infusion, animals were anesthetized with ketamine/xylazine (100 and 10 mg/kg, respectively). Chromospheres or CC were re-suspended in Optimem medium (Gibco) to a density of ~3 x 10^5^ cells in 4 μl. The cells were injected into the left striatum (+ 1.0 mm AP, ± 3.0 mm ML, −5.3 (2 μl) and −5.0 (2 μl) DV, with respect to Bregma 0). Cyclosporine A (10 mg/kg) was orally administrated daily until the end of the experiment, starting 1 day before cell transplantation. Once the injection of either 6-OHDA or cells was complete, the cannula was left in place for at least 5 min to prevent a back flow.

### Rotational behavior

To measure dopaminergic imbalance, animals were injected I.P. with amphetamine (4 mg/kg) or subcutaneously (S.C.) injected with apomorphine (2 mg/kg), as previously described [23]. The number of left and right turns was recorded for 90 min for amphetamine and 30 min for apomorphine using a custom-made computerized image- and movement- recognition system. Animals were evaluated one and two weeks after the 6-OHDA lesion and only those with > 500 ipsilateral turns induced with amphetamine or > 300 contralateral turns induced with apomorphine were used for the rest of the experiments, to ensure a high level of lesion. Grafted animals and their controls were evaluated 1, 2, 4, 6, 8 and 12 weeks post-grafting (wpg) or at equivalent times in those groups without grafts. Data is presented as the percentage of change in the number of turns after grafting relative to their initial turn number before grafting. For all the motor behavior experiments, excluding those used for analyzing the relation between graft survival and motor improvement, only grafted animals that showed a decrement ≥ 10% at 1 and/or 2 wpg were evaluated for the rest of the analyzed times.

### Beam-test for motor coordination

As previously described [24], animals were trained for 3 days to walk up 15° tilted 2 m long beams of various widths (24, 18, 12, 6 and 3 mm), which went from a starting platform up to the cage of the rats. Animals received a treat upon completion of the task during the learning period. Four days after the beginning of the training, before 6-OHDA infusion, animals were subjected to the beam test for the first time, which was followed by a second evaluation one week later. The test consisted in measuring the time it took the animals to walk from the base of the beam up to their cage at the end of the beam. Only those animals that were able to complete the test in less than 120 s during the two first evaluations were included in the experiment as either control groups or experimental groups. The values obtained from those two evaluations were set as the baseline. The test was repeated sequentially 5 times per animal, one repetition for each beam width. The order of the beams was randomly selected in each experiment. The variables that were quantified for each rat were the “total time” the animals required to reach the cage, and the time during the test while animals remained immobile (i.e. “no-movement time” variable). Animals that required more than 120 s to complete the task, or that fell off the beams, were automatically assigned a total time and no-movement time of 120 s. Animals were evaluated two times after the 6-OHDA lesion, and only those with a deficient performance (i.e. a satisfactory level of lesion) were included in the experiment as either part of the 6-OHDA group, the 6-OHDA+ chromospheres group or the 6-OHDA + vehicle group. All animals were evaluated 1, 2, 4, 6, 8 and 12 wpg or at equivalent times in those groups without grafts.

### Immunostaining

As previously described [23], animals were I.P. injected with a terminal dose of sodium pentobarbital (60 mg/kg) and were transcardially perfused with 250 ml of 0.1 M phosphate buffer, followed by 250 ml ice-cold paraformaldehyde (4% w/v in 0.1 M phosphate buffer). The brains were removed and post-fixed for 12 h in 4% paraformaldehyde. Post-fixed brain tissue was cryoprotected by incubation in 10%, 20% and 30% sucrose for 24 h for each concentration. 40 μm brain coronal sections were cut and collected in serial order and placed in 0.1 M phosphate buffer. Cultured cells were fixed in 4% paraformaldehyde in 0.1 M phosphate buffer for 30 minutes.

The following primary antibodies were used: rabbit or mouse anti-TH (1:1000, EMD Millipore), rabbit anti-DBH (1:350, Abcam). Appropriate fluorescence-tagged (Jackson ImmunoResearch Laboratories) or biotinylated (Vector Laboratories) secondary antibodies were used for microscopy visualization. For TUNEL labeling, we used the in situ cell death detection kit with TMR red (Roche), and followed the procedure for difficult tissues specified by the manufacturer.

To avoid unspecific binding, specimens were incubated for 1–2 h at room temperature in blocking solution composed of 0.1 M phosphate buffer, 0.3% Triton X-100 (Sigma-Aldrich) and either 3% normal serum (for fluorescence staining) or 2% albumin (for histochemistry staining). Specimens were incubated at room temperature in primary antibodies diluted in blocking solution. Fluorescence-tagged secondary antibodies were diluted in 0.1 M phosphate buffer and applied to the specimen for 2 h at room temperature. Biotinylated secondary antibodies were diluted in blocking solution for 2 h at room temperature. Detection of the primary-secondary biotinylated antibody complexes was achieved by peroxidase-driven precipitation of di-amino-benzidine (Sigma-Aldrich), previously amplified with streptavidin-biotin (Vector Labs)). Di-amino-benzidine labelled specimens were dehydrated in alcohol and xylene.

### Microscopy

Specimens were examined on a Leica Confocal imaging system, an Olympus FV1000 Confocal microscope, a Leica epifluorescence microscope or a Leica EZ4D microscope. For image analysis we used the Image J software [25]. All images from each sample were acquired under the same conditions (exposure, brightness, gamma levels). For graft reconstructions the photographs were taken with a Leica Confocal imaging system, and the reconstruction was performed manually only for the site of the graft.

### Cell counting for *in vitro* experiments

Samples were obtained from three independent cultures. During each culture, every adrenal gland was treated as an independent sample. CC from separate samples (n = 6) were directly seeded on treated-coverslips (poly-L-Lysine (4 μg/ml) and laminin (5 μg/ml)), whereas chromospheres from independent samples (n = 3) were first centrifuged and frozen to obtain cryo-sections of 20 μm. For each sample, a z-stack of random high-resolution images were captured at 40x magnification to obtain representative images using an Olympus FV1000 microscope. We counted the number of cells that expressed TH, DBH or both and the total number of cells marked with DAPI to identify cell nuclei. The numbers of TH^+^/DBH^-^ or TH^+^/DBH^+^ cells were normalized to the total counted nuclei (50–300 nuclei were counted per image), and are expressed as percentages from the total number of cells present in the analyzed image.

### Estimation of the number of surviving grafted cells in the striatum

**Fig 1:**
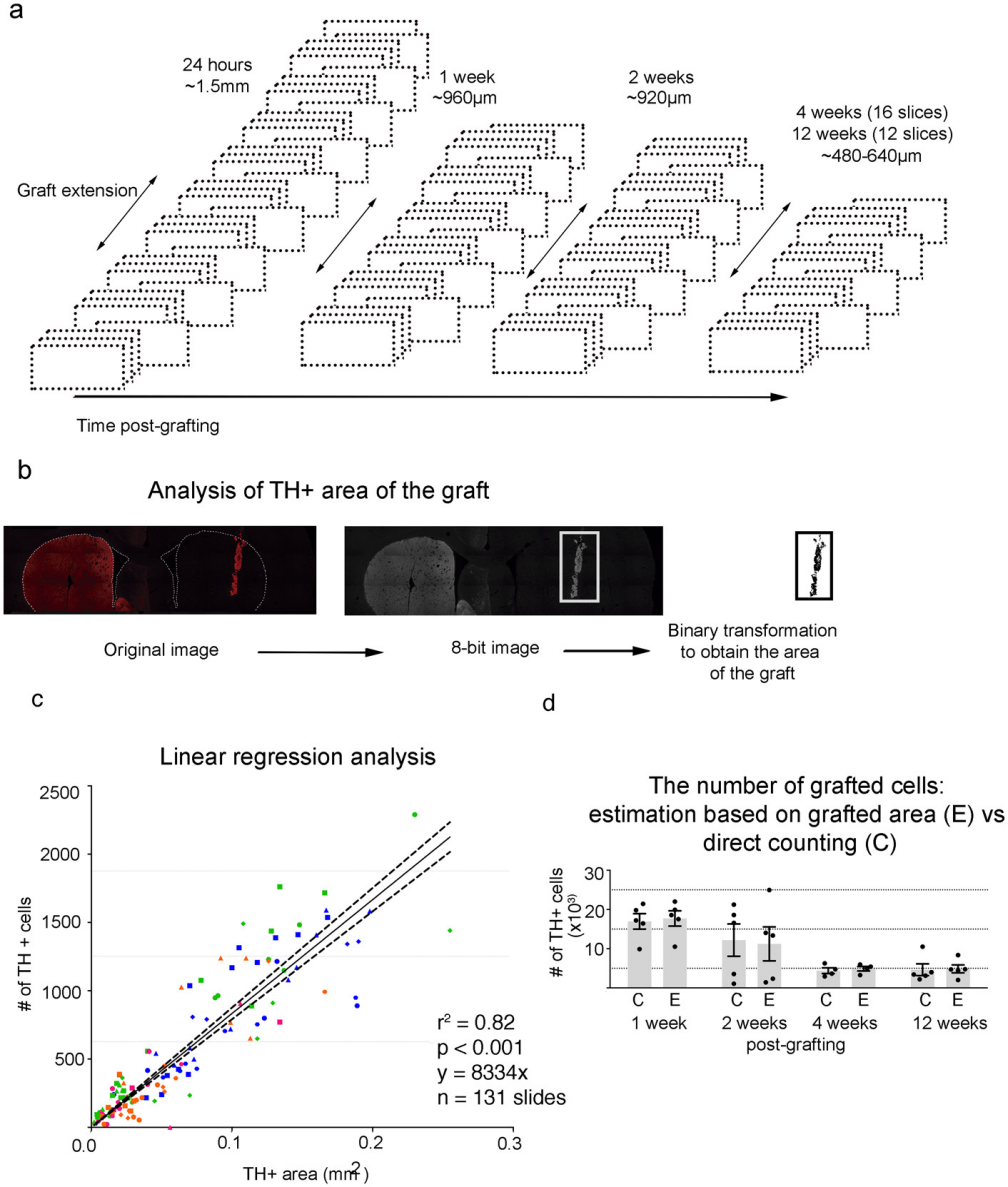
Estimation of chromosphere graft survival. (**a**) One out of four of the total coronal sections of the striatum (represented by dotted squares) were selected for immunofluorescence staining for TH. The antero-posterior extension of the graft along the striatum was estimated by determining the number of coronal sections of the striatum in which grafted cells could still be visualized, as schematized in the figure. As time after grafting progressed, the number of slices with grafted cells became smaller, indicating an antero-posterior shrinking of the grafted area due to cell death. (**b**) Quantification of the TH^+^ area of a graft. TH^+^ immunofluorescence reconstructions of the striatum (left panel) at 10x magnification were transformed into 8-bit images (middle panel), followed by binary transformation (right panel) to easily quantify the total area of the graft in each striatal section using the area quantification option in ImageJ. The insert framed by a dotted white square in the middle panel highlights the region containing the graft over which we quantified the TH^+^ area. (**c**) Graph of the estimated TH^+^ area *vs* the number of TH^+^ cells counted directly. Each data point represents the number of cells or the TH^+^ area determined for a single coronal section. Data points from grafts evaluated at 1, 2, 4 or 12 wpg are shown in different colors. The solid line is the linear regression to the data, with parameters shown at the bottom right. The dotted lines denote the uncertainty of the linear fit. (**d**) Comparison of the estimation (denoted by letter E in the graph) of graft total cell number using the extrapolation of measured TH^+^ area to the linear regression in (**c**) with the total number of TH^+^ cells directly counted (denoted by C in the graph) shows that the approximate method yields very similar results to the direct method with no statistical differences (multivariate ANOVA, P < 0.05, F = 0.0043, p = 0.9480).

To count the number of surviving grafted TH^+^ cells in the striatum, rat brains were extracted and sectioned as described earlier in the immunostaining section. Imaging analysis was done using the Image J software. The small size of the grafts (at 1, 2, 4 and 12 wpg) permitted its identification by TH immunostaining and the counting of all TH^+^ cells in each coronal section series. The small size of the graft allowed us to avoid the need for stereological counting procedures, in agreement with similar studies by other authors [26–28]. We used one out of four serial sections for quantification (Fig 1a). In cases were the graft was too small in the antero-posterior axis (i.e. only two or three sections from the selected series contained graft), we used two out of four sections to corroborate the presence of the graft and, if necessary, we used all the recollected sections. The number of TH^+^ cells inside the graft was manually quantified using z-stack images obtained with a 40x objective using confocal microscopy. Only TH^+^ cells with visible and complete nuclei were counted and the counted cell numbers were corrected by using the Abercrombie method [29]. We estimated the total number of TH^+^ cells in the striatal grafts for each animal by extrapolating the number of TH^+^ cells counted in every fourth coronal section to the rest of the sections where we did not count cells directly.

To validate the counting of TH^+^ cells as an indicator of graft survival (24 h, 1, 2 and 4 wpg), we used a TH-independent method to confirm that the TH signal was from grafted cells. To do this, chromospheres cultured for 5 days were loaded with the green fluorescent molecule called CFSE (10 μM, Life Technologies) following manufacturer’s indications for cells in suspension. After 4 days, CFSE-loaded chromospheres were grafted as described previously.

In animals used for analysis of graft survival 24 h post-grafting the grafted cells were densely packed and the size of the graft was very large, making the direct counting of cells impractical. We therefore used a different method to estimate the number of surviving cells. This consisted in estimating the number of cells using the linear relation between the TH^+^ area of the graft and the number of TH^+^ cells counted directly (Fig 1c). We obtained the TH^+^ area of the graft as follows: Reconstructions at 10x magnification were constructed using a Leica epifluorescence microscope equipped with an automatic platina. 10x reconstructions were converted first to 8-bit images and then to binary image files (Fig 1b, middle), which were then used to quantify the graft area using the area measurement function of ImageJ (Fig 1b, right). A plot of the TH^+^ area versus the number of individually counted TH^+^ cells for each striatal section from animals sacrificed at 1, 2, 4 and 12 wpg showed a good linear correlation (Fig 1c), indicating that the TH^+^ area can be reliably used to estimate the number of cells of large grafts. To verify this, we compared the total number of grafted TH^+^ cells for each grafted animal at 1, 2, 4 and 12 wpg determined by direct counting with the values obtained from our approximate method using the areas, and found that the obtained values were not statistically different (Fig 1d).

### Densitometry of dopaminergic fibers

The density of TH^+^ fibers in the striatum was calculated as previously described [30], using three to four coronal brain sections from each rat. Striatal sections were processed for TH immunohistochemistry as described above. Images were taken with a Leica EZ4D microscope with constant illumination and equal optical parameters were used for all acquired images. Digitized images were analyzed with ImageJ software. The data are presented as optical density (O.D.) values for each hemisphere. In cases where the O.D. of a lesioned striatum was higher than expected, we corroborated cell loss at the SNpc directly by counting TH^+^ cell bodies in that brain structure.

### Statistics

All group data are expressed as mean ± SEM. All statistical analyses were conducted using Prism 6 (GraphPad Software, Inc.). The tests used for each experiment are specified in each figure legend.

## Results

### Chromosphere formation in culture increases the number of dopaminergic cells and dopamine release

**Fig 2:**
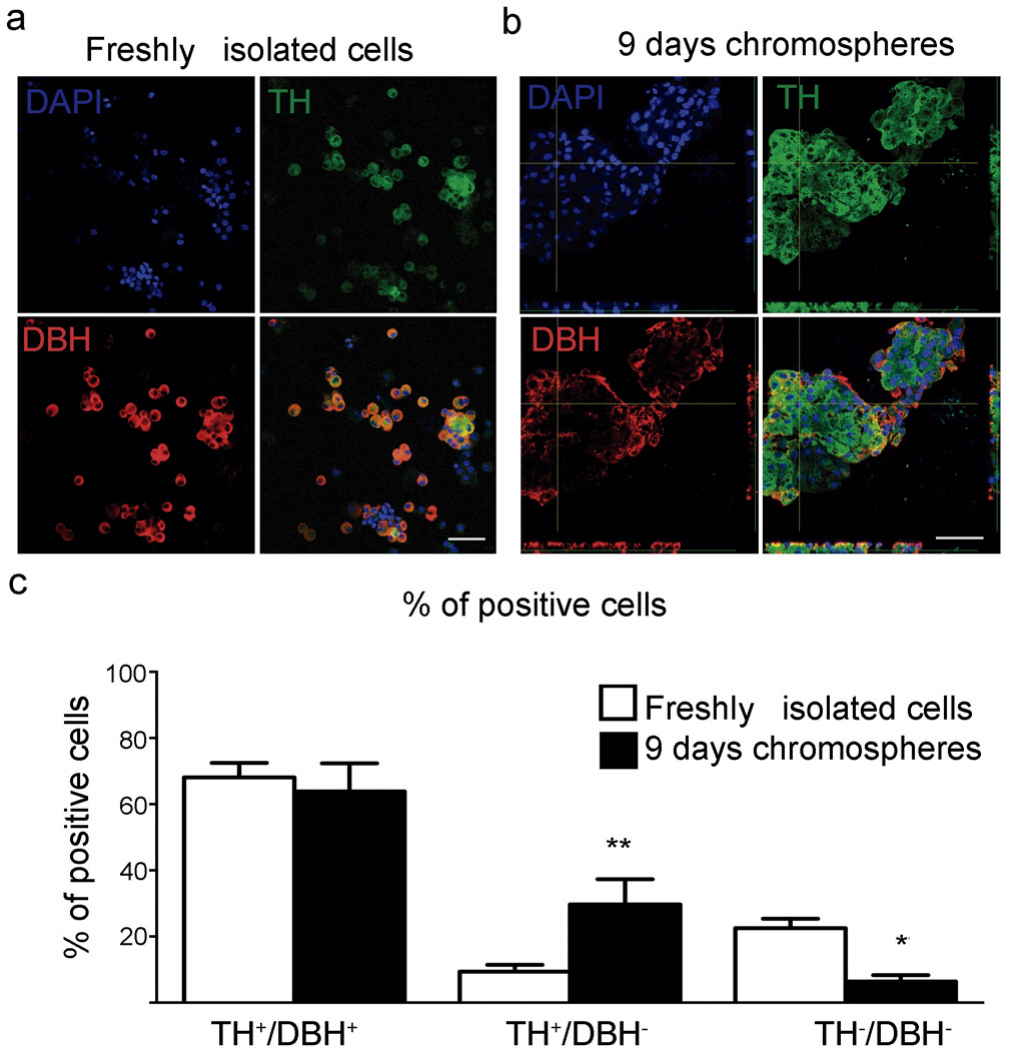
*In vitro* analysis of the number of TH^+^ cells in chromospheres and CC. (**a-b**) Confocal microscopy photographs showing double-immunofluorescence staining for TH (green, top right panel) and DBH (red, bottom left panel) in freshly isolated cells (**a**) and 9 days-chromospheres (**b**). Nuclei are marked with DAPI (blue, top left panel). Channels are shown separately and the merge of the three channels is shown at the bottom right panel. For chromosphere-slices (**b**) an orthogonal view is shown (xyz). Scale bar is 100 μm. (**c**) Percentages of the number of cells that expressed TH together with DBH (TH^+^/DBH^+^), TH only (TH^+^/DBH^-^), and cells only marked with DAPI (TH^-^/DBH^-^). The percentages were calculated relative to the total number of counted nuclei. Error bars are the SEM and the mean was obtained from three independent cultures with a total of six coverslips with fixed-freshly isolated cells or three slides with fixed-chromospheres sections. Statistically significant differences were observed for TH^+^/DBH^-^ chromospheres cells (29.71 ± 7.635%) compared to freshly isolated cells (9.363 ± 2.083%) and for TH^-^/DBH^-^ chromospheres (6.410 ± 1.937%) compared to freshly isolated cells (22.50 ± 2.9%) (multivariate ANOVA, P < 0.05; F = 71.21, DF = 2, p < 0.0001, followed by Bonferroni’s multiple comparisons *post hoc* test, p = 0.0057** for TH^+^/DBH^-^ and 0.0128* for TH^-^/DBH^-^).

A necessary feature required for the use of a cell source in PD cell replacement therapy is the ability to efficiently release dopamine. Consistently, it has been demonstrated by other authors that chromosphere formation in culture increases mRNA levels for Tyrosine Hydroxylase (TH), the rate limiting enzyme for dopamine synthesis, contrary to CC in culture, which retain their predominantly adrenergic phenotype (mRNA expression of phenylethanolamine N-methyltransferase) [20–22]. We began by determining whether chromosphere formation in culture [20–22] increases the number of dopaminergic cells relative to cultured CC from adult bovine adrenal medulla. To do this, we performed double staining immunofluorescence to detect cells that express only TH (as a marker for dopaminergic cells), or TH together with dopamine-beta-hydroxylase (DBH) (as a marker for noradrenergic and adrenergic cells) in chromospheres cultured for 9 days compared with freshly isolated cells isolated from the adrenal medulla. Notably, freshly isolated cells are expected to be composed primarily of CC, as they are the major component of the cultures after differential plating and before inducing the formation of chromospheres. Chromosphere formation in culture was observed within 3 days, and exhibited the morphological properties previously described [20–22], including their aggregation and formation of large spheres. Consistent with the previous mRNA quantifications [20–22], chromosphere formation significantly increased the number of TH^+^/DBH^-^ cells compared to freshly isolated CC (Fig 2a–2c), suggesting that a larger proportion of chromosphere cells acquire a dopaminergic phenotype.

**Fig 3:**
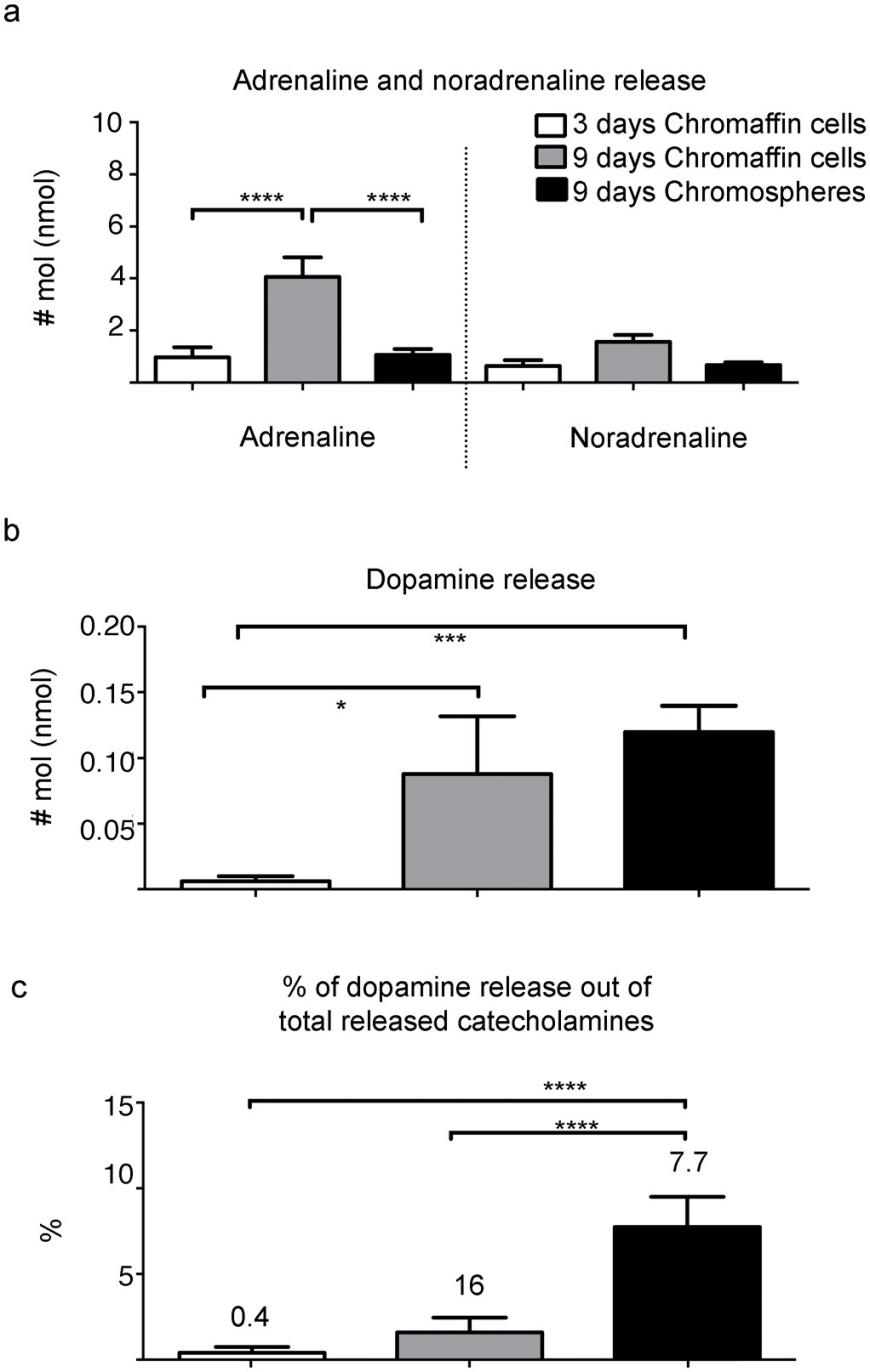
*In vitro* analysis of noradrenaline, adrenaline and dopamine release in chromospheres and CC. (**a-b**) Adrenaline, noradrenaline and dopamine release under basal conditions was determined by HPLC-ED in 3- and 9- days cultured-CC and 9 days cultured-chromospheres. 3 days CC and 9 days chromospheres were obtained from 8 independently processed adrenal glands, whereas 9 days CC were obtained from 3 independently processed adrenal glands. Data collection and analysis from cells obtained from different glands was also done separately. The extracellular medium was recollected for quantification of released catecholamines. Catecholamine quantities are expressed in nmol. (**a**) Significant differences were observed for adrenaline (3-days CC: 0.9726 ± 0.3828 nmol; 9-days CC: 4.057 ± 0.7481 nmol; 9-days chromospheres: 1.065 ± 0.2261 nmol; multivariate ANOVA, P > 0.05, F = 17.36, DF = 2, p < 0.0001; followed by Tukey´s multiple comparisons *post hoc* test, p < 0.0001****) and (**b**) dopamine (3 days CC: 0.0058 ± 0.0038 nmol; 9 days CC: 0.087 ± 0.043 nmol; 9 days chromospheres: 0.12 ± 0.019 nmol; ANOVA, P > 0.05, F = 12.53, r^2^ = 0.6103, p = 0.0005; followed by Tukey´s multiple comparisons *post hoc* test, p = 0.0145* and p = 0.0004***). (**c**) Graphic representation of the percentage of dopamine released relative to the total catecholamines released (adrenaline + noradrenaline + dopamine). Significant differences were observed between 9-days chromospheres compared to 9 and 3 days CC (ANOVA, P > 0.05, F = 27.42, r^2^ = 0.7231, p < 0.0001; followed by Tukey´s multiple comparisons *post hoc* test, p < 0.0001****).

We next proceeded to investigate whether the increase in TH^+^/DBH^-^ cells in chromospheres is accompanied by an increment in basal dopamine release relative to noradrenaline and adrenaline, and also relative to CC cultured for either 3 or 9 days. To do this, we incubated equal numbers of chromosphere cells or CC in physiological solution for 5 minutes, recollected the supernatant and analyzed the catecholamine content using HPLC-ED. For CC, adrenaline release increased significantly (~ 4-fold) between 3 and 9 days of culture (Fig 3a), while noradrenaline did not show a significant increment. In contrast, under the cell culture conditions employed for chromosphere formation, both adrenaline and noradrenaline release was similar to that of 3 days CC but adrenaline was significantly lower than that of 9 days CC (Fig 3a). On the other hand, both 9 days chromospheres and 9 days CC released significantly higher amounts of dopamine compared with 3 days CC (up to 10-fold increase in both 9 days chromospheres and CC, Fig 3b), but no significant difference in total dopamine release was found between 9 days CC and 9 days chromospheres (Fig 3b). However, the percentage of dopamine out of the total catecholamines released (adrenaline + noradrenaline + dopamine) was significantly higher in 9 days chromospheres, representing a 7.7% of the total of catecholamines, as opposed to a 1.6% in 9 days CC and 0.4% in 3 days CC (Fig 3c). Together, these results indicate that the cell culture employed for chromosphere formation maintains the initial levels of adrenaline and noradrenaline, whereas dopamine release is promoted. In contrast, CC increased both their release of adrenaline and dopamine.

### Motor behavior in 6-OHDA-lesioned rats transplanted with chromospheres

We next tested the ability of chromosphere-grafts to improve the motor alterations in 6-OHDA-lesioned animals versus non-grafted animals. In rodents, unilateral administration of 6-OHDA into the SNpc produces dopaminergic neuronal death exclusively in the brain hemisphere where the toxin was administered, providing a widely used model to study the motor symptoms of PD associated with dopamine loss in the striatum. Animals treated with 6-OHDA were grafted with chromosphere-derived cells into the lesioned-dorsal striatum 18 days after the lesion. Since a large majority of cultured chromospheres expressed TH (94 ± 2%), we used TH immonstaining to determine the location and the size of chromosphere grafts in the experiments that will follow.

**Fig 4:**
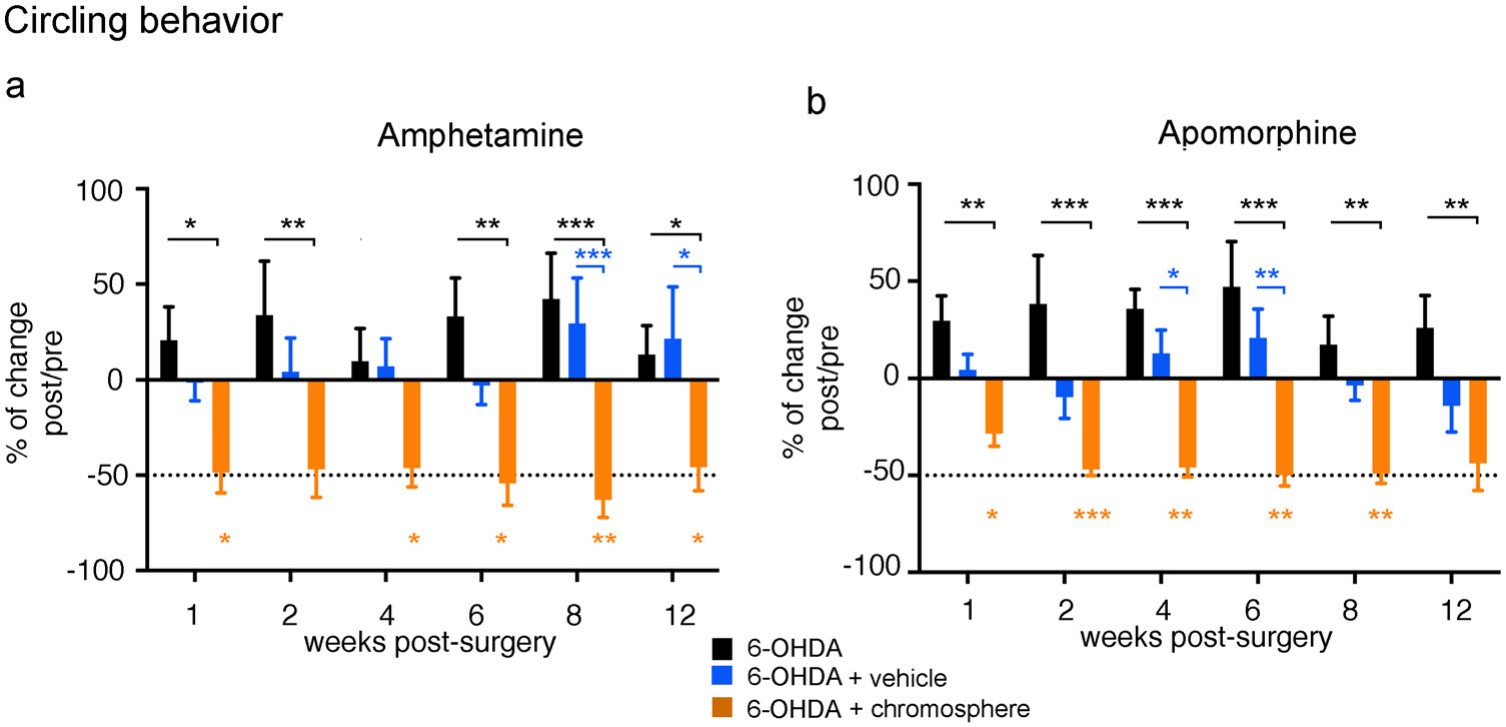
Effects of chromosphere-grafts on amphetamine and apomorphine induced circling behavior in 6-OHDA lesioned rats. (**a**) Amphetamine- and (**b**) apomorphine- induced circling behavior was evaluated in three groups at different times: 6-OHDA-lesioned (n = 6 for amphetamine and n = 6 for apomorphine, black), 6-OHDA+vehicle (n = 6 for amphetamine and n = 6 for apomorphine, blue) and 6-OHDA+chromosphere grafts (n = 7 for amphetamine and n = 6 for apomorphine, orange). For all 6-OHDA-treated animals, the number of turns after the lesion measured in the two evaluations previous to the start of the experiment (7 and 14 days after 6-OHDA administration) were used as reference point to calculate he percentage of change in the number of turns after surgery (starting at 7 days after grafting surgery). “Weeks post-surgery” denotes the time elapsed since chromospheres were surgically implanted in the test group, which was used as reference also for all other control groups. Each bar represents the mean of the percentage of change in turn number for each evaluation. The dotted horizontal line denotes a reduction in turn number of 50%. Significant differences were observed between the number of turns before and after grafting (orange asterisks) for both amphetamine-induced rotations (repeated measures ANOVA, P < 0.05, F = 7.086, DF = 6, r^2^ = 0.5415, p = 0.0035; followed by Dunnett´s multiple comparisons *post hoc* test, p < 0.05* and p < 0.01**) and apomorphine-induced rotations (repeated measures ANOVA, P < 0.05, F = 9.03, DF = 6, r^2^ = 0.569, p = 0.0128; followed by Dunnett´s multiple comparisons *post hoc* test, p < 0.05*, p < 0.01** and p < 0.001***). Also the change in turn number in the grafted group was significantly higher than in the lesioned group without graft (black asterisks) and vehicle group (blue asterisks) for both amphetamine-induced rotations (repeated measures multivariate ANOVA, P < 0.05, F = 6.991, DF = 23, p < 0.0001; followed by Tukey´s multiple comparisons *post hoc* test, p < 0.05*, p < 0.01** and p < 0.001***) and apomorphine-induced rotations (repeated measures multivariate ANOVA, P < 0.05, F = 10.36, DF = 2, p = 0.0017; followed by Tukey´s multiple comparisons *post hoc* test, p < 0.05*, p < 0.01** and p < 0.001***).

Amphetamine stimulation of animals lesioned unilaterally with 6-OHDA promotes dopamine release from all dopaminergic terminals of the non-lesioned side, inducing a stereotypical rotation movement ipsilateral to the dopamine-depleted hemisphere. Therefore, grafted cells are expected to reduce turning-behavior by releasing dopamine in the lesioned side. The control (n = 6) and sham animals (n = 6) showed no rotational behavior (data not shown), whereas the 6-OHDA-treated (n = 6) group showed a stable rotational behavior (Fig 4a and S1 Fig). In contrast, seven out of 12 grafted animals showed a reduction (i.e. a decrement ≥10%) in turn-number during the first two evaluations, which persisted in some cases for at least 3 months after grafting (Fig 4a, orange asterisks and S1 Fig). The five animals that did not show any improvement during the first two evaluations were sacrificed and were found to contain no traces of a surviving graft, as detected by TH-immunostaining, suggesting that graft survival is required for motor improvement. The highest percentage of reduction in turn-number found was 63% at 8 weeks post-grafting (wpg), with an average decrement of 51% throughout the 3 months, with a statistically significant decrement during all the evaluations (except for 4wpg) compared with the non-grafted lesioned animals (Fig 4a, black asterisks). To verify that this effect on the behavior was due to the grafted cells, and that immunosuppression and/or the vehicle administered had no effect, one additional group was added in which lesioned animals were immunosuppressed and vehicle was administered instead of chromosphere cells (6-OHDA+vehicle, n = 6). This group showed no changes in motor behavior relative to its performance before grafting and to the lesioned animals without grafts (Fig 4a and S1 Fig), confirming that the motor improvement observed in grafted animals originates from the transplanted cells.

Amphetamine-induced circling behavior is used as an indirect indicator of induced dopamine release by the grafted cells. However, that chromosphere cells can release dopamine upon stimulation by amphetamine does not indicate whether dopamine is released under basal conditions. An indirect assay to test basal dopamine release is the quantification of the turning behavior after stimulation with apomorphine, a dopamine receptor agonist. Apomorphine induces rotations contralateral to the lesioned side as a result of a sensitization of the dopamine receptors on the side lacking dopaminergic input. Thus, basal dopamine released from the grafted cells is expected to reverse sensitization and reduce the turning-behavior. Notably, six out of 10 grafted animals in apomorphine-induced rotations showed a reduction ≥10% in turn-number during the first two evaluations, compared with their number of turns before grafting (Fig 4b). The animals that presented no motor improvement in the first two evaluations were sacrificed, and shown to have no surviving TH^+^ cells. In the rest of the grafted animals the improvement persisted for at least 3 months (Fig 4b orange asterisks, and S1 Fig), although one animal was also sacrificed after 4 wpg because of an ear infection. The highest percentage of reduction in turn-number found was 50% after 6 wpg, with an average decrement of 41% during the 3 months with a statistically significant decrement during all the evaluations compared with the non-grafted animals (Fig 4b, black asterisks), and during some evaluations compared with the 6-OHDA+vehicle (Fig 4b, blue asterisks).

**Fig 5:**
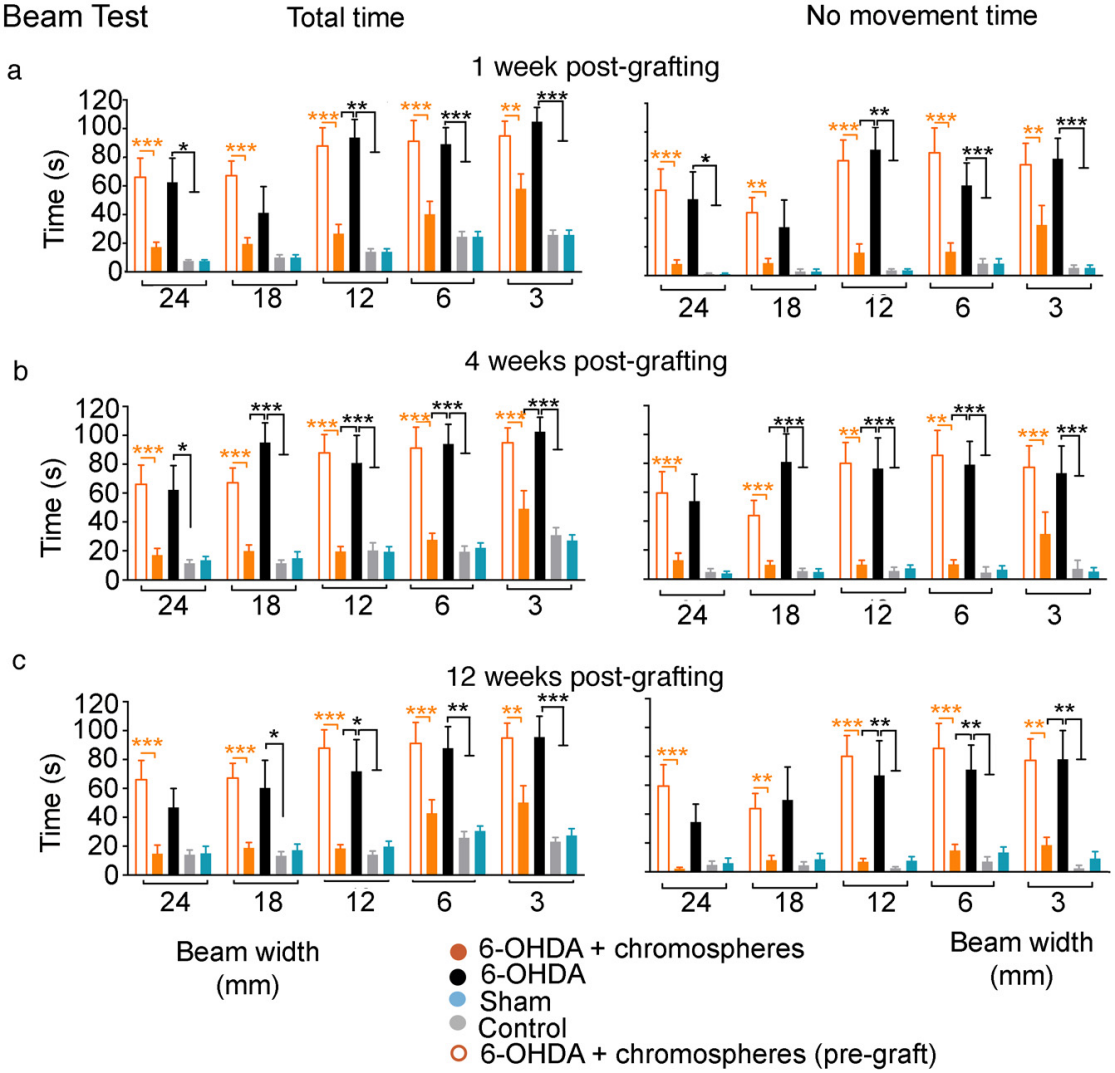
Motor performance during the beam test at 1, 4 and 12 weeks post-grafting. (**a-c, left**) The total time (seconds) that the animals took to complete the test and (**a-c, right**) the time during which the animals remained immobile (no-movement time) while the test was on-going were measured in four different groups. The performance of each animal was evaluated in all beam widths (3, 6, 12, 18 and 24 mm) in each experimental trial. The following groups were included in the experiment: control (n = 8, gray), Sham (n = 8 blue), 6-OHDA (n = 7, black), 6-OHDA + chromosphere grafts (n = 8, orange). Evaluations in all groups were carried out periodically for 3 months after the grafting surgery in the test group. Only the evaluations obtained after 1-week, 4-weeks and 12-weeks post-grafting are shown, the rest of the evaluations (2, 6 and 8 wpg) are shown in S2 Fig. Empty orange bars are the measurements from the grafted animal group obtained after the 6-OHDA-lession procedure but before grafting. Significant differences were observed between the total time and no movement time measured before grafting compared with the total time and no movement time of the same group after grafting (orange asterisks) (repeated measures multivariate ANOVA, p < 0.05, F = 5.349, DF = 4, p = 0.0018; followed by Bonferroni´s multiple comparisons *post hoc* test, p < 0.01** and p < 0.001***). Also, significant was the difference in some evaluations in both the total and no movement time between 6-OHDA lesioned animals without graft and 6-OHDA lesioned animals with chromospheres, control and sham groups (black asterisks) (repeated measures multivariate ANOVA, P < 0.05, F = 36.17, DF = 7, < 0.0001; followed by Bonferroni´s multiple comparisons *post hoc* test, p < 0.05*, p < 0.01** and p < 0.001***). Error bars are the SEM.

So far, our results with pharmacological behavioral tests show that surviving grafted chromosphere cells produce a motor improvement in the 6-OHDA model of PD, possibly by releasing dopamine. We next tested the ability of the grafts to generate motor improvements in a non-pharmacological test, which would more closely reflect the conditions where chromospheres are expected to work in the context of a PD patient. We therefore evaluated the recovery of motor alterations with the beam test, a test that measures motor coordination in terms of the total time it takes the animals to climb up tilted beams of different widths, with increasing task time denoting a poorer coordination [24]. The decrease in coordination can also be detected in the test as an increase in the time during which the animals remain immobile (i.e. ‘no movement time’ variable) [24]. During the first two weeks, we observed a reduction in the total time variable in eight out of 11 grafted animals relative to their evaluations before grafting. The three animals that presented no motor improvement were sacrificed and shown to have a negligible level of surviving TH^+^ cells. For the rest of the evaluations we observed a statistically significant reduction in the total time in all the evaluations of 6-OHDA+chromospheres animals (n = 8) compared with their performance before grafting (Fig 5 and S2 Fig, orange asterisks). The observed improvement persisted for at least 3 months after grafting and no differences were observed when compared to control and sham groups (Fig 5c). The 6-OHDA group (n = 7) showed more difficulties to perform the test over the 3 months of evaluation (Fig 5 and S2 Fig), whereas the grafted animals presented a clear reduction in the total time and no movement time that was statistically significant in some evaluations when compared with lesioned animals without graft (Fig 5 and S2 Fig, black asterisks). These data suggest that animals with surviving chromosphere grafts improve their motor coordination evaluated with a non-pharmacological test.

In the three behavioral tests, we found that the grafted animals that did not present an improvement in the first two evaluations, which we used as exclusion criterion, had negligible levels of graft survival. To validate the hypothesis that the grafts are responsible for the observed motor improvement in the animals included in the experiment, we performed a histological analysis in all animals used in the behavioral experiments to test for both dopaminergic denervation in the lesioned striatum and for survival of the grafted cells at 12 wpg. The analysis showed that for the three behavioral tests, all animals that presented a robust and consistent motor improvement also had surviving grafted TH^+^ cells in the striatum, whereas animals that did not show a consistent improvement had no surviving grafts (see Table 1, top panel, as summary of the behavioral results together with the percentages of motor improvement and the degree of graft survival for all experimental animals used for behavioral evaluation).

**Table 1:** Survival and motor efficacy of chromosphere cell grafts.

Experiment	No. of animals at the beginning of the experiment (A)	No. of animals with motor improvement during the first 2 weeks (B)	% of motor efficacy ([B x 100] / A)	No. of animals with graft at the end of the experiment (C)	% of survival efficacy ([C x 100] / A)
1. Circling behavior	12 and 10	7 and 6	59	5 and 3	36
2. Beam Test	11	8	73	6	55
**Total (1 and 2)**	33	21	77	14	42
3. Relation between survival and motor improvement	13	6	46	8	62
**Total (1–3)**	46	27	59	22	47

This table is a summary of the motor and survival efficacy of chromosphere grafts obtained from all the experiments performed where motor behavior was evaluated. The percentage of motor efficacy ([B x 100] / A) was calculated as the number of animals with motor improvement during either 1 or 2 wpg (B) from the number of animals originally grafted (A). The percentage of survival efficacy ([C x 100] / A) was calculated as the number of animals with surviving grafted cells at the end of the experiment (C) from the number of animals originally grafted (A).

**Fig 6:**
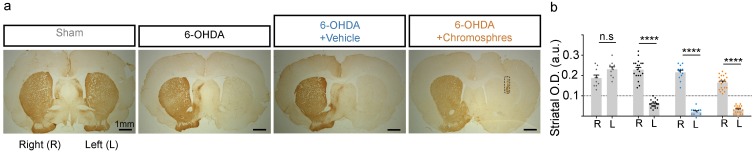
Density of dopaminergic fibers in the striatum of all animals used for long-term behavioral evaluations. The degree of dopaminergic denervation in the striatum was measured to corroborate the effective destruction of the dopaminergic terminals. (**a**) Representative photographs of TH immunohistochemistry in striatal coronal sections obtained with a stereo-microscope for the experimental and control groups denoted in the figure. (**b**) Optical density of TH^+^ signal for the two hemispheres was determined separately from three striatal sections per animal for all the animals used in the long-term behavioral experiments described thus far (i.e. those evaluated up to 12 wpg). The data in the graph correspond to the following experimental groups: Sham (n = 10 randomly selected animals, gray dots), 6-OHDA (n = 19, black dots), 6-OHDA + vehicle (n = 12, blue dots), 6-OHDA + chromospheres (n = 21, orange dots). For animals with chromosphere grafts, we excluded from the densitometry analysis the TH^+^ area that corresponds to the graft (**a**, indicated by a dotted box). R, right hemisphere; L, left hemisphere. Significant differences were observed only between the lesioned (left) and non-lesioned (right) hemispheres in animals treated with 6-OHDA (multivariate ANOVA, P < 0.05, F = 27.89, DF = 6, p < 0.0001; followed by Tukey´s multiple comparisons *post hoc* test, P < 0.0001****). Error bars are the SEM. Scale bar is 1 mm.

The results observed in the behavioral tests suggests that surviving chromosphere grafts induce motor improvement. However, the motor improvement observed could be the result of a partial rather than a complete loss of dopaminergic terminals in the lesioned striatum. To rule this out, we determined using TH immunohistochemistry the O.D. of striatal TH^+^ fibers in both hemispheres for all the lesioned animals assayed in behavioral experiments, excluding the TH^+^ area derived from the graft in transplanted animals (Fig 6a). We found that the degree of dopaminergic fiber loss in the lesioned striatum was similar in all the analyzed groups treated with 6-OHDA, corresponding to a severe dopaminergic denervation of the terminals in the striatum, and no significant differences were found between any of the lesioned experimental groups (Fig 6b). Thus, the recovery observed in the behavioral tests is consequence of the grafted cells rather than a lower difference in the degree of the lesion induced by the neurotoxin. Moreover, the lack of TH^+^ staining in the lesioned striatum of grafted animals also indicates that the grafts did not produce a motor recovery by promoting sprouting of surviving dopaminergic terminals from the host. Together, our results suggest that grafted chromospheres could release dopamine and possibly other molecules (e.g. noradrenaline and adrenaline) under basal conditions and after pharmacological stimulation, and that these released molecules influence the striatal circuit to promote a motor recovery.

### Survival and TH expression of chromospheres grafted in a 6-OHDA rat model of PD

**Fig 7:**
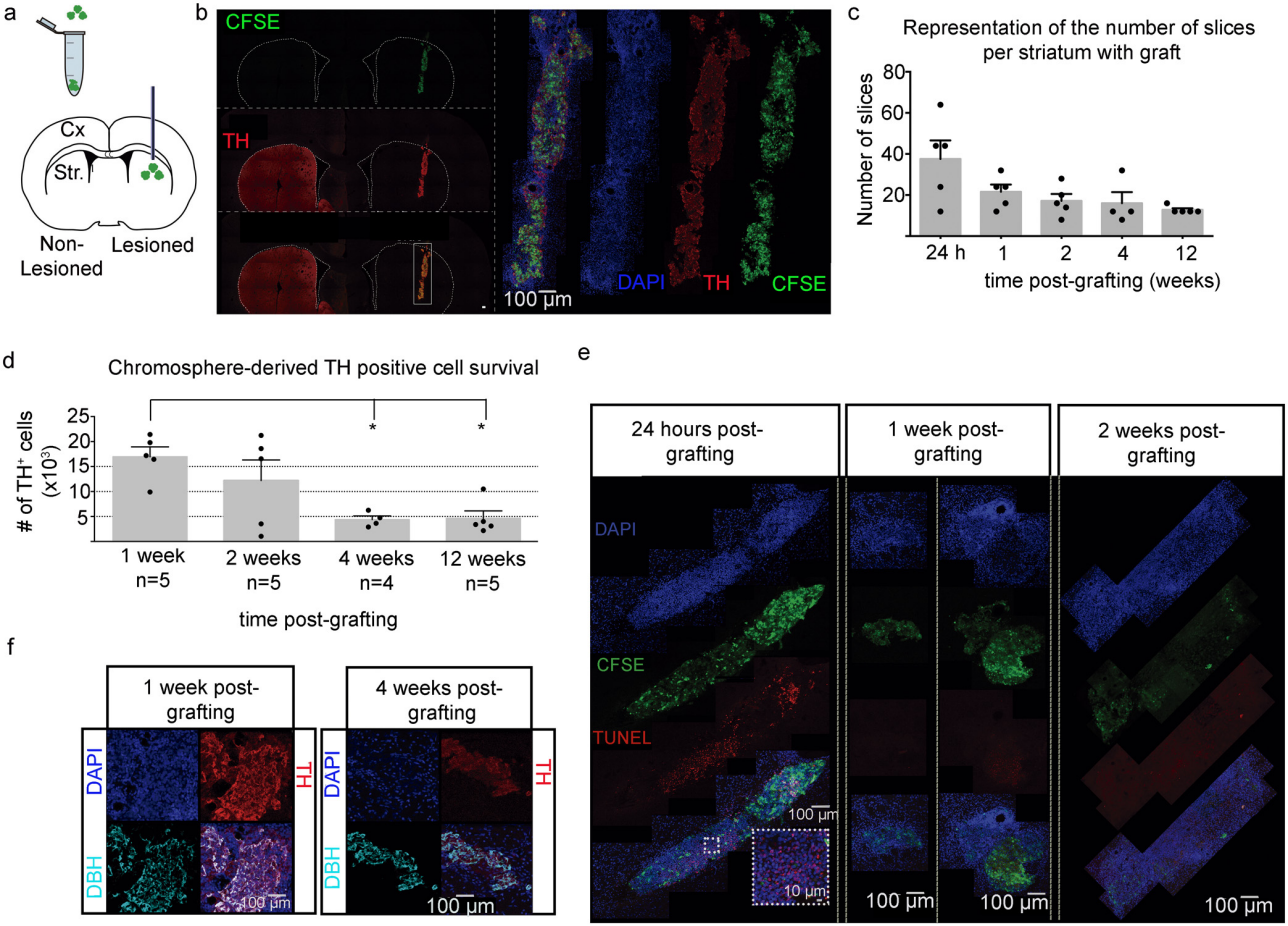
Survival and TH expression of chromospheres grafted into the striatum of 6-OHDA lesioned rats. (**a**) Schematic representation of the grafting site. Chromosphere cells were loaded with a green fluorescent dye (CFSE) before grafting, and stereotaxically implanted into the lesioned striatum (Str). Cx (cortex). (**b**, left panel) Representative reconstruction of a coronal section of the striatum from a 6-OHDA-lesioned animal with grafted chromospheres after immunofluorescence staining for TH (red). Both lesioned (right) and non-lesioned (left) hemispheres are shown. The top panel shows CFSE fluorescence (green), the middle panel shows TH immunofluorescence and the lower panel is the merge. Images at 10x magnification were obtained with an epifluorescence microscope. The reconstruction was done automatically using an automatic platina. The scale bar is 100 μm. (**b**, right panel) Representative reconstruction at higher magnification (40x) of a chromosphere graft obtained from the same coronal section as in the left panel using a confocal microscope. The reconstruction was done manually. DAPI (blue), TH immunostaining (red) and CFSE (green) are shown in separate channels and are merged in the top-left panel. (**c**) The number of coronal sections of the striatum found to contain grafted cells for each animal decreases during the first 24 h post-grafting. Striatal coronal sections of 40 μm were recollected in serial order and every fourth section was used for TH immunostaining or every two sections for those cases where the graft was smaller along the antero-posterior axis. 24 h (n = 5); 1, 2, 12 wpg (n = 5); 4 wpg (n = 4). (**d**) The TH^+^ surviving-grafted cells were counted directly from images obtained with 40x objective at 1, 2, 4 and 12 wpg (values obtained: 16 955 ± 1 978, 12 195 ± 4 129, 4 391 ± 729, 4 639 ± 1 497, respectively). Statistical differences are denoted by asterisks (ANOVA, P > 0.05, F = 5.685, r^2^ = 0.5321, p = 0.0083; followed by Tukey´s multiple comparisons *post hoc* test, P < 0.05*). (**e**) Representative confocal microscopy reconstructions showing grafted apoptotic cells marked with TUNEL (red) in coronal slices of the grafted striatum of animals at 24 h, 1 or 2 wpg. Grafted cells are shown in green as detected by CFSE fluorescence, and DAPI is shown in blue. The lower panel shows the overlay. All images were acquired using the same microscope settings and a 40x objective. Reconstructions of the grafted area were performed using a confocal microscope as described in (**b**). The insert framed by a dotted white square was obtained at 63x magnification from the section denoted by the dotted square in the image at lower magnification. (**f**) Representative double-immunofluorescence staining for TH (red) and DBH (cyan) in coronal sections from the striatum of two grafted animals at 1 and 4 wpg.

Given the variability in graft survival observed from the previous assays, we decided to directly quantify the capacity of chromosphere-grafts to survive in host animals as a function of time while using an additional detection method that is TH-independent. This is especially important since our previous assays were limited to the detection of TH^+^ cells as an indicator for graft survival. Animals treated with 6-OHDA were grafted with chromosphere-derived cells previously loaded with the fluorescent dye CFSE (n = 26, Table 2, top panel). The CFSE molecules allowed us to corroborate that the observed TH^+^ cells were transplanted cells instead of endogenous cells, as the CFSE-containing cells were clustered tightly in a region consistent with the location of the graft and also co-localize with TH (Fig 7b). TH^+^ cells were quantified in independent groups of grafted animals sacrificed at 1, 2 or 4 wpg (for short-term evaluation). We also included five randomly selected animals (from those with surviving cells at 12 wpg) from the 3 month-behavioral experiments described above to compare survival at 12 wpg with survival at earlier time-points. The grafted area was easily detected in the dorsal striatum by the presence of TH^+^ cells (and CFSE for the groups sacrificed at 1, 2 or 4 wpg) (Fig 7b), indicating the survival of the cells. Of the total grafted animals, 19 out of 26 showed CFSE or TH^+^ cells in the grafted area (Table 2, top panel). We proceeded to estimate the number of surviving TH^+^ cells in those animals with a detectable graft. In serial sections of the striatum of grafted animals, the grafts were found to extend across 8 to 64 slices (Fig 7c) of the host striatum, which is equivalent to a cross-section of 0.6–1.5 mm of the host striatum. Importantly, the number of slices in which the graft could be visualized decreased with the time after grafting, indicating a progressive reduction in the number of grafted cells. The presence of TH^+^ cells was restricted to the region of the graft, and no processes were found outside this region. Additionally, contrary to the *in vitro* assays where 26% of the analyzed cells showed a dopaminergic phenotype (TH^+^/DBH^-^), here almost all TH^+^ cells were also positive for DBH (Fig 7f), suggesting a predominantly adrenergic/noradrenergic phenotype.

**Table 2:** Survival efficacy of chromosphere cell grafts.

Experiment	No. of animals at the beginning of the experiment (A)	No. of animals with graft at the end of the experiment (B)	% of survival efficacy ([B x 100] / A)	Decrement of TH^+^ cells grafted compared with the initial number of grafted cells (%)
**Survival**				
24 h	6	5	83	84
1 wpg	7	5	71	94.3
2 wpg	7	5	71	95.9
4 wpg	6	4	67	98.5
**Total**	26	19	73	
**Behavior (circling behavior and beam test)**^a^				
12 wpg	33	14	42	98.5
**Relation between survival and motor improvement**^a^				
4 wpg	13	8	62	
**Grand Total**	72	41	57	

This table is a summary of the survival efficacy of chromosphere grafts obtained from all the experiments performed. The percentage of survival efficacy ([B x 100] / A) was calculated as the number of animals with surviving grafted cells at the end of the experiment (B) from the number of animals originally grafted (A). The percentage of decrement of TH^+^ cells in the graft was determined only for the animals in the top and middle panels. We used the initial estimated number of grafted cells (~3 x 10^5^ cells) to calculate the percentage of decrement in surviving cells at 1, 2, 4 and 12 wpg.

^a^These data is from animals used to evaluate motor behavior (Table 1).

The total number of TH^+^ cells was only significantly smaller at 4 and 12 wpg when compared with 1 wpg (Fig 7d). However, the data shows that the percentage of decrement in TH^+^ cells between 1 wpg and 2 wpg was about 29%, followed by a 64% of decrement between 2 wpg and 4 wpg. No additional significant loss was observed between 4 and 12 wpg. These data suggest that cellular death is more prominent during the first weeks after transplantation, so that one additional group was added to estimate the number of TH^+^ cells 24 h after grafting. In this group we estimated that the number of TH^+^ cells was about 48 ± 12 x10^3^ (S3 Fig), which is much higher than the number of surviving cells even at 1wpg. These data indicate that the highest decrement of TH^+^ cells occurred during the first 24 h post-grafting, with a decrement of 84% from the initial 3 x 10^5^ chromosphere-derived grafted cells (Table 2, top panel). We finally corroborated the cell death was more prominent in the first week after grafting by using TUNEL labeling to determine the apoptotic cellular death in the short-term (24 h post grafting, 1 and 2 wpg), and found more intense TUNEL labeling 24 h post grafting as compared to samples obtained from brains at 1 or 2 wpg (Fig 7e).

### Chromosphere-cell survival is not the only requirement for improving motor alterations

**Fig 8:**
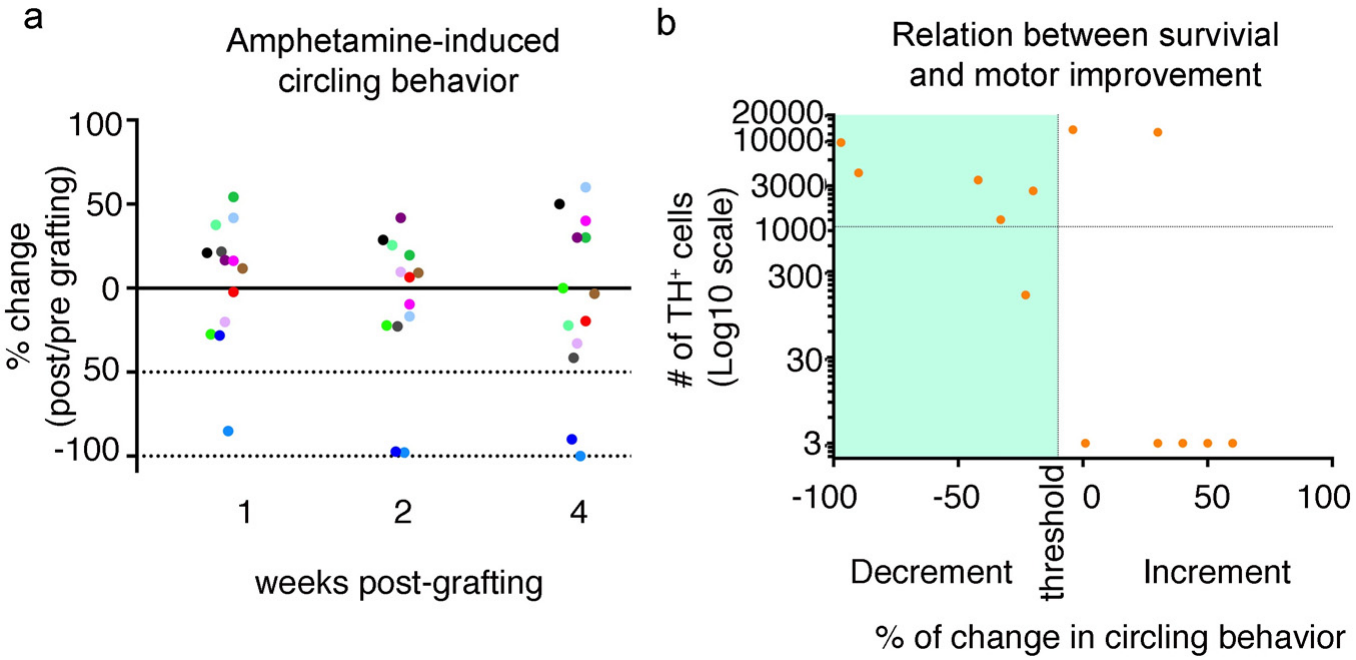
Relation between chromosphere survival and motor improvement. Amphetamine-induced circling behavior was used to determine whether there is a relation between chromosphere survival and reduction in turn number in grafted animals. (**a**) Percentage of change in amphetamine induced circling behavior in 6-OHDA-lesioned animals grafted with chromospheres. Each animal is represented by a different dot color. The percentage of change was calculated using the initial number of turns for each 6-OHDA-treated animal before grafting as reference, so that negative values denote a decrease in the number of turns, whereas positive values denote an increase. The dotted horizontal lines denote a reduction in turn number of 50% and 100%. At the end of the experiment 6 out of 13 animals reduced their number of turns by ≥ 10%. Animals were sacrificed after the last evaluation and the number of surviving grafted cells were determined by direct counting. (**b**) Relation between the percentage of change in turn number at 4 wpg relative to the number of turns before grafting (x-axis) and the number of estimated surviving cells at 4 wpg (y-axis) for chromosphere-grafted animals used for the behavioral experiments in (**a**). The graph quadrant corresponding to a reduction in turn number is highlighted in teal. Each dot represents data from a single animal. The obtained relation shows that all animals with a reduction in turn number have surviving grafted cells, and that, with two notable exceptions, animals without changes in turn number did not present any surviving grafted cells.

Our findings in the behavioral experiment showing that graft survival within the first weeks after transplantation was determinant for observing motor improvement indicates that the grafts are required for establishing a functional effect. However, our exclusion protocol for these experiments prevented us from directly comparing the relation between graft survival and motor recovery over a longer period of time for all the animals that we grafted, regardless of whether they presented an improvement in the first two evaluations. In an effort to directly assess the relation between survival and motor recovery over a longer period of time, we transplanted an independent group of lesioned animals and determined the decrement in amphetamine-induced circling behavior at at 1, 2 and 4 wpg. At the end of the experiment (4 wpg) animals were sacrificed to determine the survival of TH^+^ cells to correlate this data with the behavioral results. At 1 and 2 wpg six out of 13 animals decreased their number of turns by ≥ 10% compared with their number of turns before grafting. However, only four of those animals maintained the motor improvement until 4 wpg. Two additional animals exhibited decreased turn number only at 4 wpg (Fig 8a). Thus, a total of six animals reduced their turn number at the end of the experiment, from which four animals showed a high level of graft survival, with 2.6 to 9.5 x 10^3^ TH^+^ cells, and the other two animals presented 1 208 and 160 surviving cells (Fig 8b). On the other hand, five out of seven of the animals that did not show any motor improvement presented a negligible level of graft survival (Fig 8b), consistent with our previous observations (Table 1). However, it is important to note that two animals that did not show any motor improvement had more than 12 x 10^3^ TH^+^ cells, which is comparable to the graft survival observed in the animals with robust and consistent motor improvement (Fig 8b). This important observation indicates that cell survival is not the only requirement for inducing motor improvement.

### Comparison between chromosphere- and chromaffin-grafts in a 6-OHDA rat model of PD

**Fig 9:**
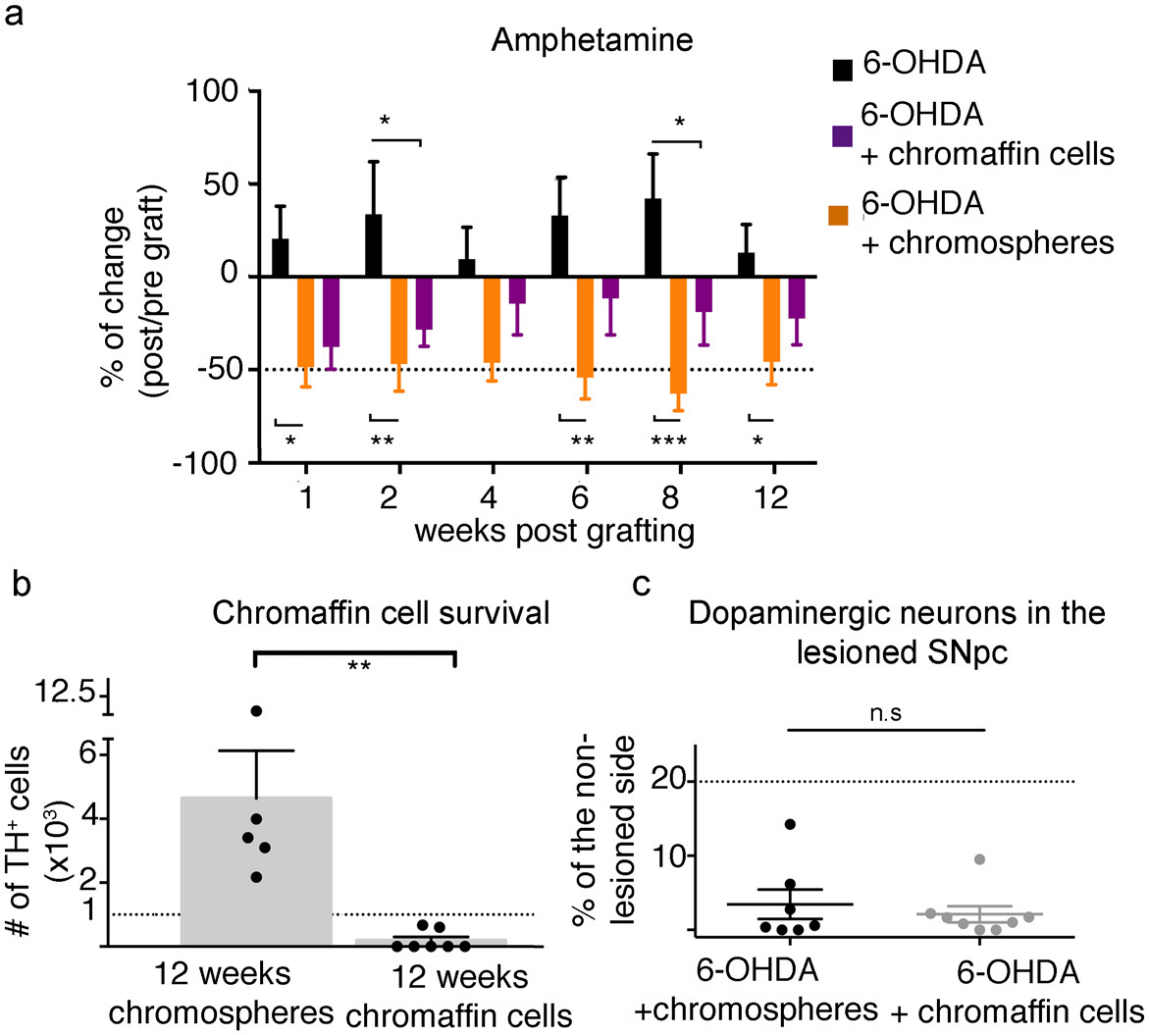
Comparison between chromosphere grafts and CC grafts. (**a**) Percentage of change in circling behavior induced by amphetamine in 6-OHDA lesioned animals with either chromosphere (n = 7, orange) or CC (n = 8, purple) grafts and in a 6-OHDA lesioned group (n = 6). Each bar represents the mean ± SEM. The dotted horizontal line denotes a reduction in turn number of 50%. Significant differences in chromaffin grafted animals were observed at 2 and 8 wpg when compared to the lesioned group without graft (repeated measures multivariate ANOVA, P < 0.05, F = 6.991, DF = 23, p < 0.0001; followed by Tukey´s multiple comparisons *post hoc* test, p < 0.05*, p < 0.01** and p < 0.001***) (black asterisks), but not when compared with their circling behavior before grafting (repeated measures ANOVA, F = 1.142, r^2^ = 0.1402, p = 0.3485). (**b**) The survival of chromaffin grafted cells was determined by directly counting TH^+^ cells in immunostained coronal brain slices. The dots represent the number of surviving grafted cells from single animals, and the bars denote the mean ± SEM. The dotted horizontal line represents the number of cells equivalent to 1 x 10^3^. A significant difference was observed between chromaffin and chromosphere cell survival (Unpaired t-test, P > 0.05, t = 3.572, df = 10, p = 0.0051**). (**c**) Percentage of dopaminergic cells in the lesioned SNpc in relation to the non-lesioned hemisphere in animals unilaterally lesioned with 6OHDA and grafted with either CC (grey) or chromospheres (black). The number of TH^+^ cells in the SNpc of both the lesioned and non-lesioned sides was determined by direct cell counting of TH immunostained brain slices. Data was normalized to the number of counted TH^+^ cells in the non-lesioned SNpc. Each data point represents an individual animal, and the mean ± SEM is indicated by solid horizontal lines. The dotted horizontal line denotes a loss of 20% of TH^+^ cells in the lesioned hemisphere compared to the non-lesioned SNpc. No significant differences were observed (two-tailed unpaired t-test) between animals grafted with either chromospheres or CC, suggesting that the degree of lesion induced by the 6-OHDA toxin was similar in all the analyzed animals.

So far, we have shown that in some grafted animals chromosphere cells are able to survive and to produce from moderate to robust motor improvements in both pharmacological and non-pharmacological behavioral tests. We have also shown that survival is necessary to produce improvement, but is not the only factor determining graft functionality. Given the progenitor-like properties of chromospheres [19–22], we were interested in determining whether they exhibit better properties as those of differentiated CC in the context of PD. To test this, we grafted 6-OHDA-lesioned animals with CC obtained from 3 days cultures and tested for their ability to reduce turning behavior after treatment with amphetamine, as done previously for chromosphere grafts. These results were compared with our data from amphetamine-induced circling behavior in chromosphere grafted animals and 6-OHDA lesioned animals evaluated during 12 wpg (Fig 4a). Eight out of 10 chromaffin-grafted animals showed a reduction in turn-number during the first two evaluations, with a mean of 40% decrement during the first evaluation (similar to chromosphere-grafted animals) and a 27% mean decrement during all the 3 months, which is lower than the reduction in chromosphere-grafted animals (Fig 9a and S4 Fig). The decrement in turn number was statistically different at 2 and 8 wpg when the turn numbers of animals grafted with CC were compared with lesioned animals without grafts, but not when compared with their circling behavior before grafting (Fig 9a). These results suggest that chromosphere grafts produce a more robust and consistent improvement in motor alterations as compared with CC grafts.

The lower motor improvement produced by CC grafts could be due to lower long-term survival of the grafts, deficient dopamine release and/or other dopamine-independent mechanisms. In order to determine whether CC survive less than chromosphere-derived cells, we quantified chromaffin graft-survival at 12 wpg using the animals from the behavioral experiment described above, and compared these results with our data for chromosphere grafts at 12 wpg (Fig 7d). Calculating the average number of surviving CC at 12 wpg only for the few animals with surviving cells, as we had previously done for chromospheres, suggests that chromosphere-grafted cells had a ~7.3-fold higher survival than chromaffin-grafted cells, with an average number of 634 ± 34 considering only the two animals with a surviving CC graft. However, one out of the 8 chromaffin-grafted animals was not included in the analysis as it exhibited an unusually large number of surviving grafted cells (~30 000 TH^+^ cells, larger than in all other grafted animals at 12 wpg and at other analyzed times). Notably, this animal presented a reduction in turning behavior of 47% at the end of the experiment (12 wpg). This reduction is only 1.7-fold better than the average value of the whole population of chromaffin grafted animals (27%), and not better than the mean of decrease observed in chromosphere-grafted animals (51%, Fig 9a). We also quantified the degree of dopaminergic denervation at the level of the SNpc in the 8 chromaffin-grafted animals used for the experiments described above, and we detected an almost complete lesion (lesioned SNpc had only 0–10% of the TH^+^ cells present in the non-lesioned side). A similar degree of lesion in the SNpc of the 6-OHDA-treated animals that were used in the chromosphere-graft experiments (Fig 9c) was found. These results indicate that the loss of dopaminergic neurons within the SNpc induced by 6-OHDA was comparable in all the experiments in this study.

In addition, even in a short-term analysis (4 wpg), we observed that only three out of 12 animals grafted with CC presented low graft survival (0.20, 1.7 and 1.9 x 10^3^ TH^+^ cells respectively, with an average number of 1 267 ± 536), whereas the rest of the animals presented a negligible level of graft survival (data not shown).

## Discussion

In the present study we determined the capacity of chromospheres to survive and to induce motor improvements in 6-OHDA-lesioned animals. We showed that chromospheres survive in ~57% of grafted animals. Also, when chromosphere grafted cells survived, they induced statistically significant motor improvements in an array of behavioral tests. In addition, we also showed that compared to CC, chromosphere-cells survive for longer periods of time and in higher numbers. Chromosphere grafts also induced a more consistent motor improvement than CC based on our own results and on similar measurements of motor improvement by CC grafts reported by others [5,31,32]. In animals with surviving grafted cells, chromosphere survival was found to be 3.5- and 7-fold greater than CC in both short- (4 wpg) and medium-term (12 wpg), respectively. Notably, the survival of chromospheres detected in this study is slightly higher that the survival of CC measured in other studies where CC´s were either transdifferentiated with neuronal growth factor (NGF) [33,34], grafted together with implanted pumps for NGF delivery [35], or co-grafted with NGF producing cells [33,36,37]. The former procedures produced only a 4-fold higher survival than untreated CC.

On the other hand, previous studies have shown that fetal mesencephalic cells and dopaminergic neurons derived from embryonic stem cells have a significantly better survival than CC after being grafted into PD animal models. In this regard, fetal mesencephalic cells have exhibited a survival of 3–5% out of the total grafted cells [8], which is higher than our 1.5% survival of chromosphere-cells at 12 wpg (equivalent to ~4 638 TH^+^ cells). Dopaminergic neurons derived from stem cells, on the other hand, have exhibited a survival of 2.7% [38], 4.1% [39] or 6% [40], and have been evaluated in some cases for longer periods of time than the three months evaluated in this study. In addition, both fetal mesencephalic cells and dopaminergic cells derived from stem cells have shown to induce a good motor recovery with a minimal number of surviving cells: ~657 fetal mesencephalic cells achieved ~50% of reduction in apomorphine-induced turns in the 6-OHDA model [41], and ~986 dopaminergic neurons derived from human stem cells were reported to achieve a complete reduction in turns induced by amphetamine [42]. The reported functional potency of chromospheres was > 40% in amphetamine-induced rotations, with a minimal requirement of 3 498 TH^+^ surviving cells (Fig 8), although we did observe in some animals that have > 4 300 surviving cells a reduction in turn number of almost 100%.

Although chromosphere survival and functionality was lower than what has been obtained using fetal mesencephalic cells and dopaminergic neurons from embryonic stem cells, it is important to note that when chromosphere grafts survive they actually produce significant motor improvements in the three behavioral tests used in this study. The properties of the chromospheres could be potentially improved through different culture manipulations. As an example, chromosphere cells can be transformed into a more neuron-like morphology in culture [22,43], which could potentially enable them to extend projections and to better integrate into the host circuit after grafting. This could be relevant as the lack of innervation of the host striatum by the grafted chromospheres observed in the present work could be a contributing factor to their lower functional potency.

The difference observed between the survival of CC and chromospheres could be due to the latter deriving from the expansion and aggregation of progenitor-like cells, together with the changes induced by the chromosphere culture protocol [19–22]. Evidence supporting this idea has been obtained by different approximations. First, chromosphere cells possess the ability of self-renewal, as they form clonal-secondary spheres [20]. Second, the chromosphere culture protocol has been shown to increase the expression of neural stem cell markers such as Nestin, CD133, Notch-1, Notch-2, Hes-1, NGF receptor, Musashi-1, Snai-2, Sox-9, Sox-10 among others, detected at the mRNA level by using RT-PCR [19–22] and microarrays [44]. Notably, CC do not express these markers or express them at very low levels [20,21]. The mechanisms underlying these changes in expression are not clear, but the natural environment in which adrenal medulla cells are located contributes to their protein expression pattern [45]. Local factors released from the adrenal cortex (e.g. steroid hormones and growth factors) influence CC differentiation and proliferation [46,47]. Thus, the isolation of adrenal medulla cells together with the culture conditions employed for chromosphere formation could contribute to the enrichment and expansion of chromaffin progenitor-like cells and the increment in neuronal progenitor markers. bFGF, which was included in the culture medium used to induce formation of chromospheres, could be playing a determinant role. This mitogenic factor has been shown to increase the number of dopaminergic neurons derived from cultured fetal mesencephalic cells [48], and has been used in several protocols for the *in vitro* differentiation of human embryonic stem cells into dopaminergic neurons, and to support the survival of the cells [49]. Addition of bFGF to CC cells in culture has been shown to induce a neuronal trans-differentiation and to promote the outgrowth of processes. However, the changes induced by bFGF are smaller than those produced by NGF [50]. Also important is that trans-differentiated CC appear to have difficulties to survive for more than 6 days in bFGF containing-medium [50], contrary to chromospheres that can be maintained by several weeks [20].

An additional and important issue that should be noted is that regardless of the medium-term survival observed in chromosphere grafts, there was a large population of animals (43% out of all grafted animals) in which the graft did not survive (Table 2, bottom panel). The absence of grafted cells in some of the animals could be related to the implantation procedure, as one out of 6 animals sacrificed 24 h after grafting did not exhibit any grafted-cells (Table 2, top panel). Nevertheless, it is likely that other factors could be involved. In this regard, the immune system could be playing an important role in the rapid and effective clearance of the graft observed during the first week, which was equivalent to a 95% decrement from the initial number of grafted cells. If this were true, improving the immunosuppression regimen could enhance the long-term survival of chromospheres and increase the number of animals in which the grafts survive at all. Accordingly, Jensen et al [51] reported that the oral administration of the immunosuppressant cyclosporine, used in the present study, is less effective for preventing graft rejection than its parenteral administration. However, our results also showed that survival of the grafted chromosphere cells is not the only factor involved in generating a functional recovery, as some animals presented no motor improvements despite having equivalent levels of graft survival as animals that presented robust motor improvements. Thus, in order to provide a predictable and positive outcome for any type of graft it is important to identify the sources of such experimental variability.

Finally, it must be mentioned that the mechanism by which grafted chromospheres are able to produce motor improvements is unclear. Speculatively, both pharmacological tests used in this study indirectly suggest that the grafts release dopamine, as both assays are directed towards uncovering imbalances in dopamine neurotransmission between the lesioned and the intact hemispheres by specifically promoting dopamine release or by activating dopamine receptors. In the beam test it has also been shown that the administration of drugs that increase the dopaminergic transmission in the striatum of aged rats (e.g. levodopa and amphetamine) leads to improved performance [24], suggesting that the release of dopamine also plays an important role in the outcome of this non-pharmacological test. However, other substances released by the grafts could also contribute to the functional recovery. Among those substances, noradrenaline and adrenaline could also be playing an important role as they have been shown to activate dopaminergic receptors [52,53], and are released by chromospheres in much larger quantities than dopamine when assayed *in vitro* (Fig 3). It is important to note that as a consequence of dopaminergic-receptor hypersensitivity in the lesioned side [54–56], low quantities of dopamine released by the grafts could be sufficient to enhance behavioral responses associated with dopamine-function. In addition, it must be mentioned that chromospheres are composed of a heterogeneous population of cells and that other factors not related to catecholamines could contribute to the mechanism responsible for the functional improvement caused by the grafts. In early transplantation studies using adrenal and extra-adrenal derived CC, it was shown that the sprouting of surviving host dopaminergic fibbers caused by neurotrophic factors (e.g. glial cell derived neurotrophic factor) released by the grafted cells was a major determinant for the functional effect of the grafts [8]. However, in the present study we did not observe TH immunoreactivity outside the grafted area (Fig 6), suggesting that chromosphere grafted cells are not inducing a neuroprotective action on the host dopaminergic fibbers, at least under our experimental conditions. However, this observation does not rule out a role for other non-catecholamine molecules in producing the motor improvements caused by the grafts, and more studies are necessary in this regard.

This is the first study that determines the potential use of chromospheres as a cell replacement candidate in PD. Further experiments will be necessary to optimize the functional outcome and survival of this cell source. These include grafting cultured dopaminergic-like neurons derived from chromospheres [22], grafting cultured chromospheres derived from a more homogeneous cell population, grafting specific cell types derived from chromosphere (e.g. TH^+^/DBH^-^ cells), or optimizing the immunosuppression regimen. Other important aspects that require further investigation are the mechanisms responsible for the motor improvement as a consequence of the grafts including the role of other substances (such as growth factors), the electrophysiological properties of the grafted cells and the relation between the release of dopamine and other substances with the motor improvement.

## Supporting Information

S1 Fig Individual data from circling behavior induced by amphetamine and apomorphine. (**a**) Amphetamine- and (**b**) apomorphine- induced circling behavior was evaluated in three groups at different times: 6-OHDA lesioned (n = 6 for amphetamine and n = 6 for apomorphine, black), 6-OHDA+vehicle (n = 6 for amphetamine and n = 6 for apomorphine, blue) and 6-OHDA+chromosphere grafts (n = 7 for amphetamine and n = 6 for apomorphine, orange). For all 6-OHDA-treated animals, the number of turns after the lesion measured in the two evaluations previous to the start of the experiment (7 and 14 days after 6-OHDA administration) were used as reference for calculating the percentage of change in the number of turns in the evaluations after surgery (starting at 7 days after grafting). “Weeks post-surgery” denotes the time elapsed since chromospheres were surgically implanted in the test group. Each data point represents the percentage of change in turn number for a single animal after one evaluation, and the lines represent the mean of each group for each evaluation. The dotted line denotes no change (0%).
(TIF)

S2 Fig Motor performance during the beam test at 2, 6 and 8 weeks post-grafting. (**a-c, left**) The total time (seconds) that the animals took to complete the test and (**a-c, right**) the time during which the animals remained immobile (no-movement time) while the test was on-going were measured in four different experimental groups. The performance of each animal was evaluated in all beam widths (3, 6, 12, 18 and 24 mm). The following groups were included in the experiment: control (n = 8, gray), Sham (n = 8 blue), 6-OHDA (n = 7, black), 6-OHDA + chromosphere grafts (n = 8, orange). Evaluations in all groups were carried out periodically for 3 months after the grafting surgery. Only the evaluations obtained after 2-weeks, 6-weeks and 8-weeks post-grafting are shown. Empty orange bars are the measurements from the grafted animal group obtained after the 6-OHDA-lession procedure but before grafting. Significant differences were observed between the total time and no movement time measured before grafting and the total time and no movement time of the same group after grafting (orange asterisks) (repeated measures multivariate ANOVA, p < 0.05, F = 5.349, DF = 4, p = 0.0018; followed by Bonferroni´s multiple comparisons *post hoc* test, p < 0.01** and p < 0.001***). Also, significant was the difference in some evaluations in both the total and no movement time between 6-OHDA lesioned animals without graft and 6-OHDA lesioned animals with chromospheres, control and sham groups (black asterisks) (repeated measures multivariate ANOVA, P < 0.05, F = 36.17, DF = 7, < 0.0001; followed by Bonferroni´s multiple comparisons *post hoc* test, p < 0.05*, p < 0.01** and p < 0.001***). Error bars are the SEM.
(TIF)

S3 Fig Survival of chromospheres grafted into the striatum of 6-OHDA lesioned rats at 24 h post-grafting. The TH^+^ surviving-grafted cells were counted manually from images obtained with a 40x objective (at 1, 2, 4 and 12 wpg) or estimated from the total TH^+^ immunostained area from 10x reconstructions (24 h post-grafting). No statistical analysis was performed to compare survival after 24h with 1–12 wpg, since we used different quantification methods, but an almost 3-fold higher number of TH^+^ cells at 24 h post-grafting compared to 1 wpg can be observed.
(TIF)

S4 Fig Individual data of amphetamine circling behavior of chromosphere and CC grafted animals. Circling behavior induced by amphetamine was evaluated in 6-OHDA lesioned animals with chromaffin (n = 8, purple) and chromosphere (n = 7, orange) grafts at 12 wpg. The percentage of change in turn number was calculated relative to the number of turns before grafting for each individual animal. Each data point represents the percentage of change in turn number for a single animal after one evaluation, and the lines represent the mean of each group for each evaluation. The dotted line denotes no change (0%).
(TIF)
