# Supplementary figures and images for: Intrastriatal Grafting of Chromospheres: Survival and Functional Effects in the 6-OHDA Rat Model of Parkinson's Disease
Source: PLoS One. 2016 Aug 15;11(8):e0160854. doi: 10.1371/journal.pone.0160854 (PMC4985142; doi:10.1371/journal.pone.0160854)

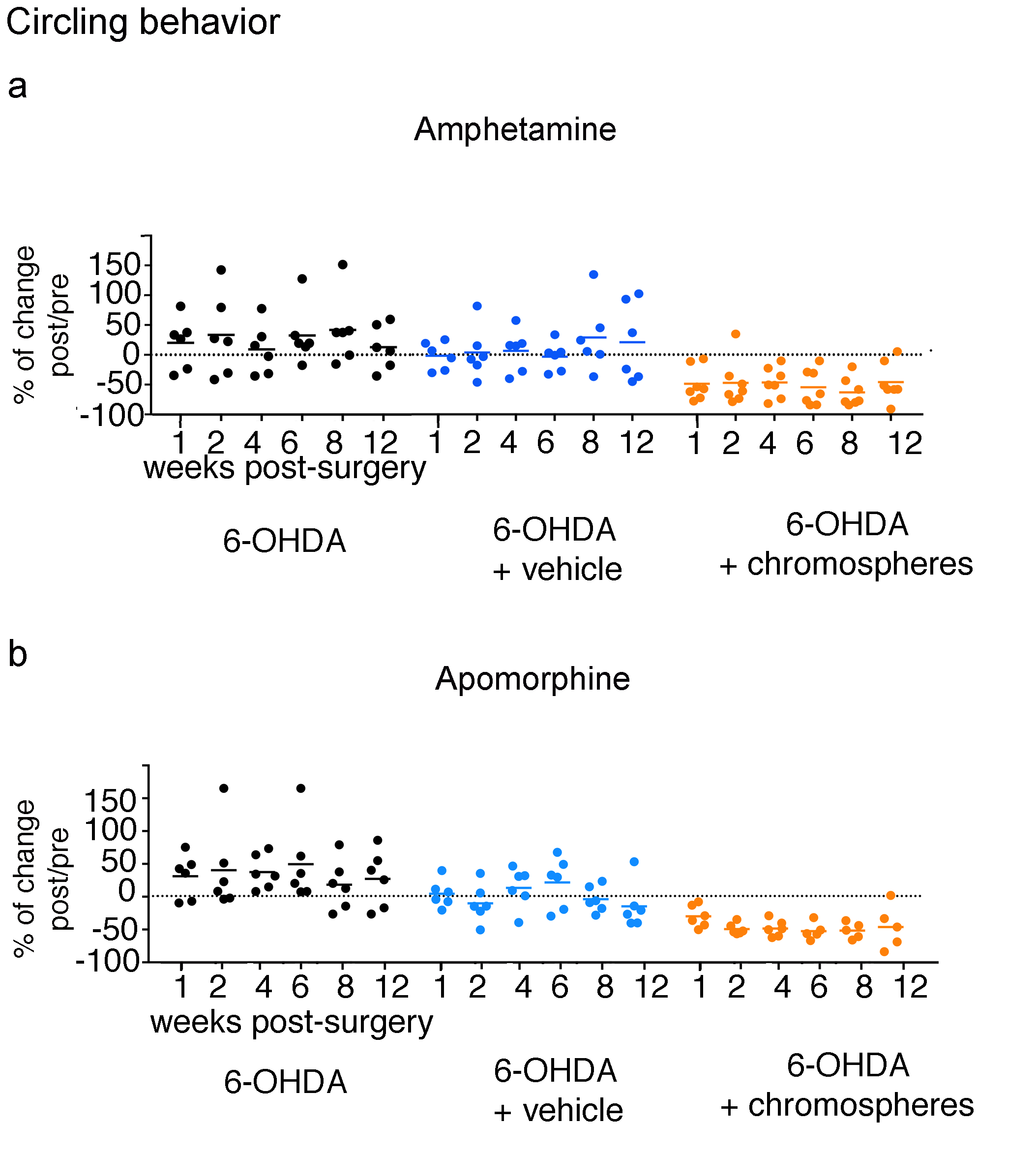

Supplement: S1 Fig — (a) Amphetamine- and (b) apomorphine- induced circling behavior was evaluated in three groups at different times: 6-OHDA lesioned (n = 6 for amphetamine and n = 6 for apomorphine, black), 6-OHDA+vehicle (n = 6 for amphetamine and n = 6 for apomorphine, blue) and 6-OHDA+chromosphere grafts (n = 7 for amphetamine and n = 6 for apomorphine, orange). For all 6-OHDA-treated animals, the number of turns after the lesion measured in the two evaluations previous to the start of the experiment (7 and 14 days after 6-OHDA administration) were used as reference for calculating the percentage of change in the number of turns in the evaluations after surgery (starting at 7 days after grafting). “Weeks post-surgery” denotes the time elapsed since chromospheres were surgically implanted in the test group. Each data point represents the percentage of change in turn number for a single animal after one evaluation, and the lines represent the mean of each group for each evaluation. The dotted line denotes no change (0%). (TIF) [file pone.0160854.s001.tif]

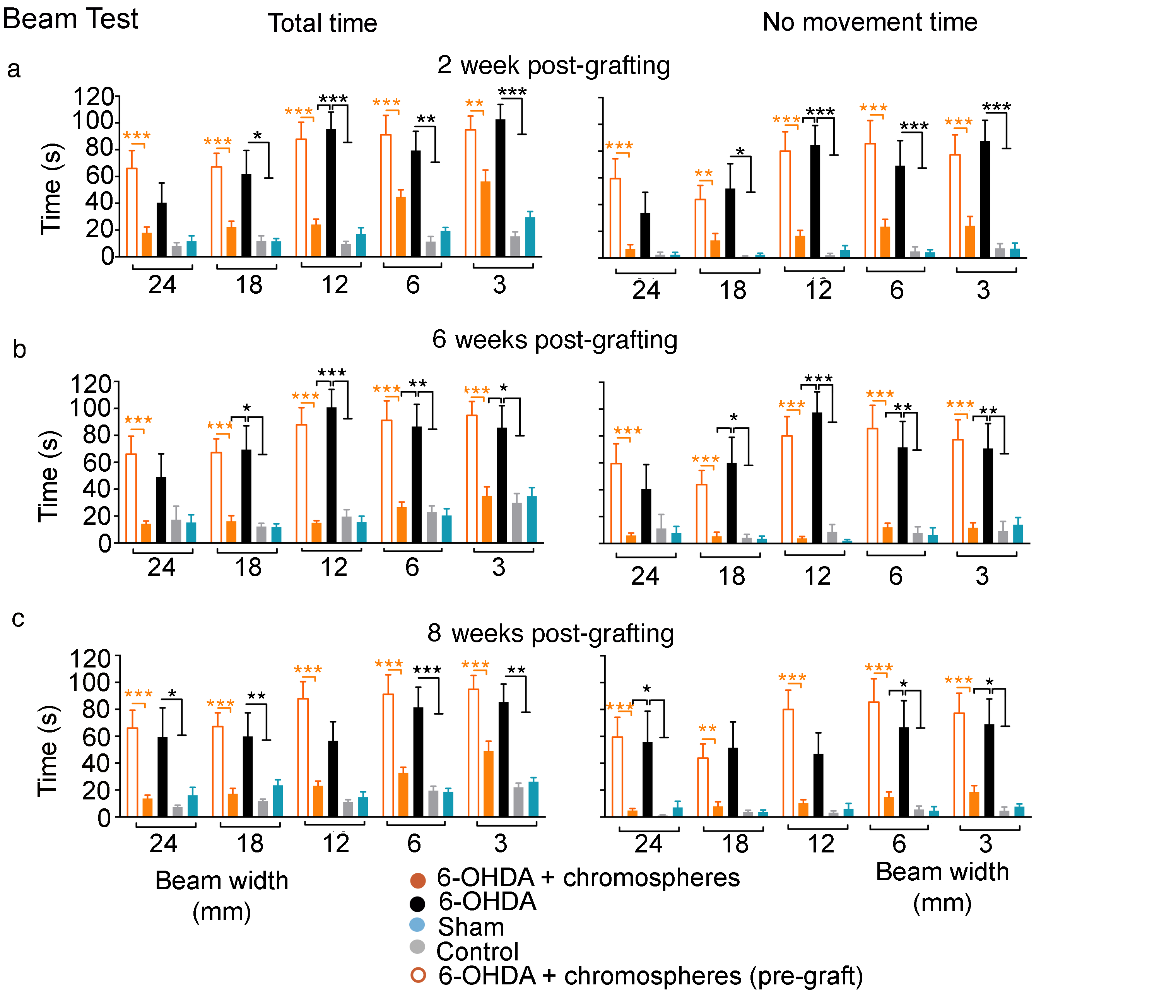

Supplement: S2 Fig — (a-c, left) The total time (seconds) that the animals took to complete the test and (a-c, right) the time during which the animals remained immobile (no-movement time) while the test was on-going were measured in four different experimental groups. The performance of each animal was evaluated in all beam widths (3, 6, 12, 18 and 24 mm). The following groups were included in the experiment: control (n = 8, gray), Sham (n = 8 blue), 6-OHDA (n = 7, black), 6-OHDA + chromosphere grafts (n = 8, orange). Evaluations in all groups were carried out periodically for 3 months after the grafting surgery. Only the evaluations obtained after 2-weeks, 6-weeks and 8-weeks post-grafting are shown. Empty orange bars are the measurements from the grafted animal group obtained after the 6-OHDA-lession procedure but before grafting. Significant differences were observed between the total time and no movement time measured before grafting and the total time and no movement time of the same group after grafting (orange asterisks) (repeated measures multivariate ANOVA, p < 0.05, F = 5.349, DF = 4, p = 0.0018; followed by Bonferroni´s multiple comparisons post hoc test, p < 0.01** and p < 0.001***). Also, significant was the difference in some evaluations in both the total and no movement time between 6-OHDA lesioned animals without graft and 6-OHDA lesioned animals with chromospheres, control and sham groups (black asterisks) (repeated measures multivariate ANOVA, P < 0.05, F = 36.17, DF = 7, < 0.0001; followed by Bonferroni´s multiple comparisons post hoc test, p < 0.05*, p < 0.01** and p < 0.001***). Error bars are the SEM. (TIF) [file pone.0160854.s002.tif]

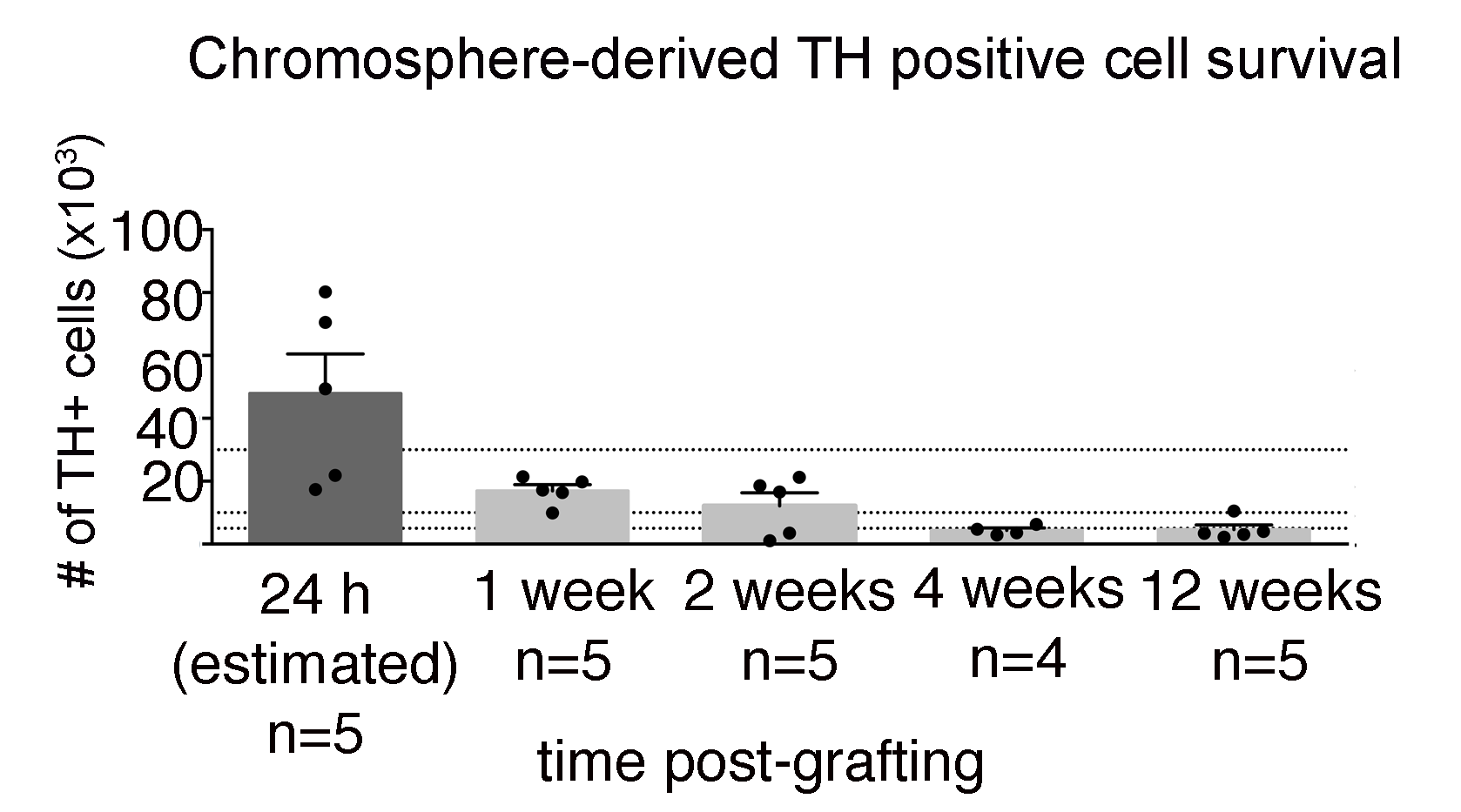

Supplement: S3 Fig — The TH+ surviving-grafted cells were counted manually from images obtained with a 40x objective (at 1, 2, 4 and 12 wpg) or estimated from the total TH+ immunostained area from 10x reconstructions (24 h post-grafting). No statistical analysis was performed to compare survival after 24h with 1–12 wpg, since we used different quantification methods, but an almost 3-fold higher number of TH+ cells at 24 h post-grafting compared to 1 wpg can be observed. (TIF) [file pone.0160854.s003.tif]

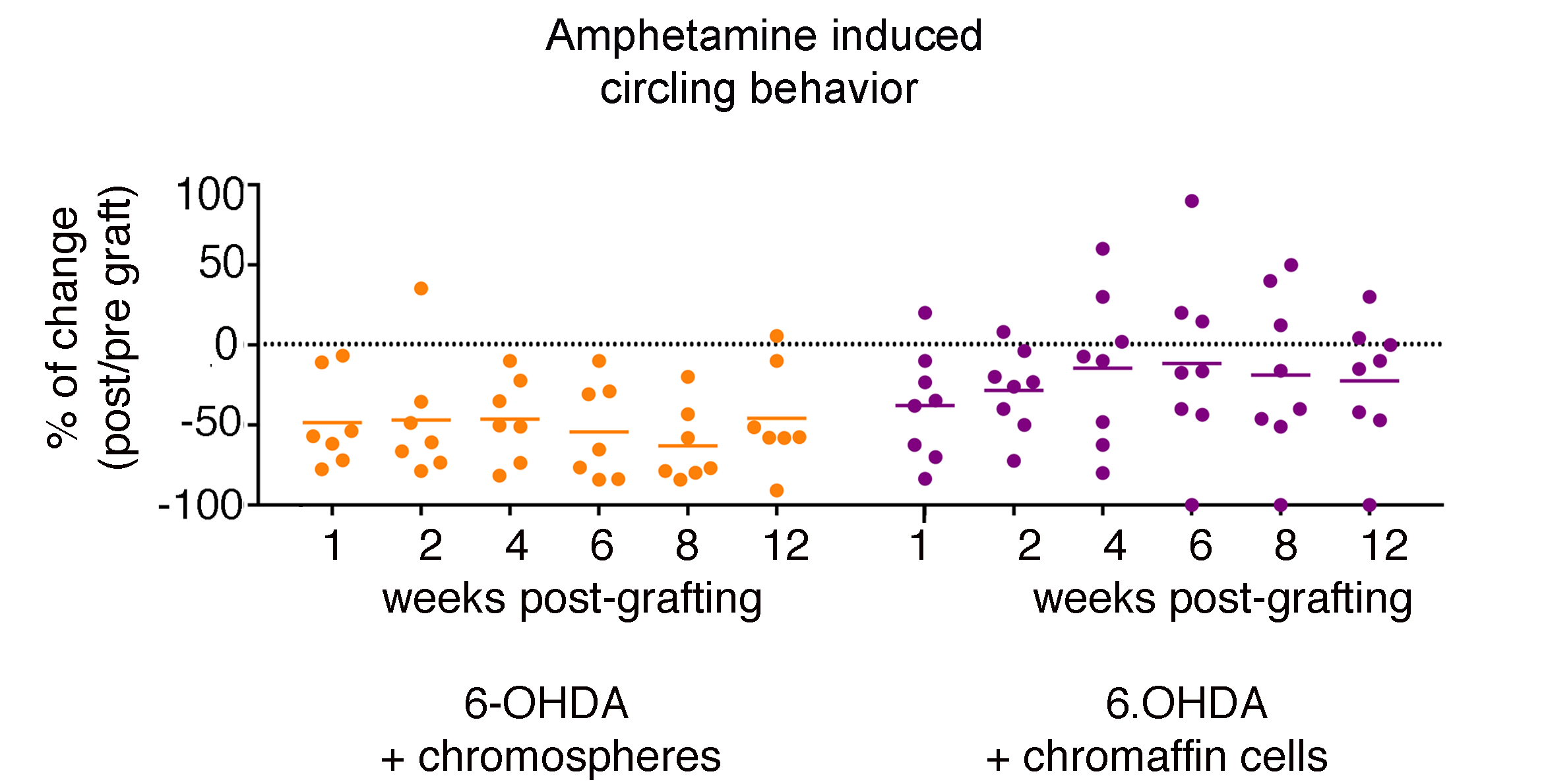

Supplement: S4 Fig — Circling behavior induced by amphetamine was evaluated in 6-OHDA lesioned animals with chromaffin (n = 8, purple) and chromosphere (n = 7, orange) grafts at 12 wpg. The percentage of change in turn number was calculated relative to the number of turns before grafting for each individual animal. Each data point represents the percentage of change in turn number for a single animal after one evaluation, and the lines represent the mean of each group for each evaluation. The dotted line denotes no change (0%). (TIF) [file pone.0160854.s004.tif]
